# Consumer Health Search on the Web: Study of Web Page Understandability and Its Integration in Ranking Algorithms

**DOI:** 10.2196/10986

**Published:** 2019-01-30

**Authors:** Joao Palotti, Guido Zuccon, Allan Hanbury

**Affiliations:** 1 Qatar Computing Research Institute Doha Qatar; 2 Institute for Information Systems Engineering Technische Universität Wien Vienna Austria; 3 University of Queensland Brisbane Australia; 4 Complexity Science Hub Vienna Vienna Austria

**Keywords:** readability, literacy, comprehension, patients, machine learning

## Abstract

**Background:**

Understandability plays a key role in ensuring that people accessing health information are capable of gaining insights that can assist them with their health concerns and choices. The access to unclear or misleading information has been shown to negatively impact the health decisions of the general public.

**Objective:**

The aim of this study was to investigate methods to estimate the understandability of health Web pages and use these to improve the retrieval of information for people seeking health advice on the Web.

**Methods:**

Our investigation considered methods to automatically estimate the understandability of health information in Web pages, and it provided a thorough evaluation of these methods using human assessments as well as an analysis of preprocessing factors affecting understandability estimations and associated pitfalls. Furthermore, lessons learned for estimating Web page understandability were applied to the construction of retrieval methods, with specific attention to retrieving information understandable by the general public.

**Results:**

We found that machine learning techniques were more suitable to estimate health Web page understandability than traditional readability formulae, which are often used as guidelines and benchmark by health information providers on the Web (larger difference found for Pearson correlation of .602 using gradient boosting regressor compared with .438 using Simple Measure of Gobbledygook Index with the Conference and Labs of the Evaluation Forum eHealth 2015 collection).

**Conclusions:**

The findings reported in this paper are important for specialized search services tailored to support the general public in seeking health advice on the Web, as they document and empirically validate state-of-the-art techniques and settings for this domain application.

## Introduction

### Background

Search engines are concerned with retrieving relevant information to support a user’s information-seeking task. Commonly, signals about the topicality or aboutness of a piece of information with respect to a query are used to estimate relevance, with other relevance dimensions such as understandability and trustworthiness [1] being relegated to a secondary position or completely neglected. Although this might be a minor problem for many information-seeking tasks, there are some specific tasks in which dimensions other than topicality have an important role in the information seeking and decision-making process. The seeking of health information and advice on the Web by the general public is one such task.

A key problem when searching the Web for health information is that this can be too technical, unreliable, generally misleading, and can lead to unfounded escalations and poor decisions [2-4]. Where correct information exists, it can be hard to find and digest among the noise, spam, technicalities, and irrelevant information. In *high-stakes search tasks* such as this, access to poor information can lead to poor decisions, which ultimately can have a significant impact on our health and well-being [4,5]. In this study, we are specifically interested in the understandability of health information retrieved by search engines and in improving search results to favor information understandable by the general public. We leave addressing reliability and trustworthiness of the retrieved information to future work; however, this can be achieved by extending the framework we investigate here.

The use of general purpose Web search engines such as Google, Bing, and Baidu for seeking health advice has been largely analyzed, questioned, and criticized [6-11], despite the commendable efforts these services have put into providing increasingly better health information, for example, the Google Health Cards [12].

Ad hoc solutions to support the general public in searching and accessing health information on the Web have been implemented, typically supported by government initiatives or medical practitioner associations, for example, HealthOnNet.org (HON [13]) and HealthDirect.gov.au, among others. These solutions aim to provide *better* health information to the general public. For example, HON’s mission statement is “to guide Internet users to reliable, understandable, accessible and trustworthy sources of medical and health information.” On the contrary, do the solutions that these services currently employ actually provide this type of information to the health-seeking general public?

**Figure 1:**
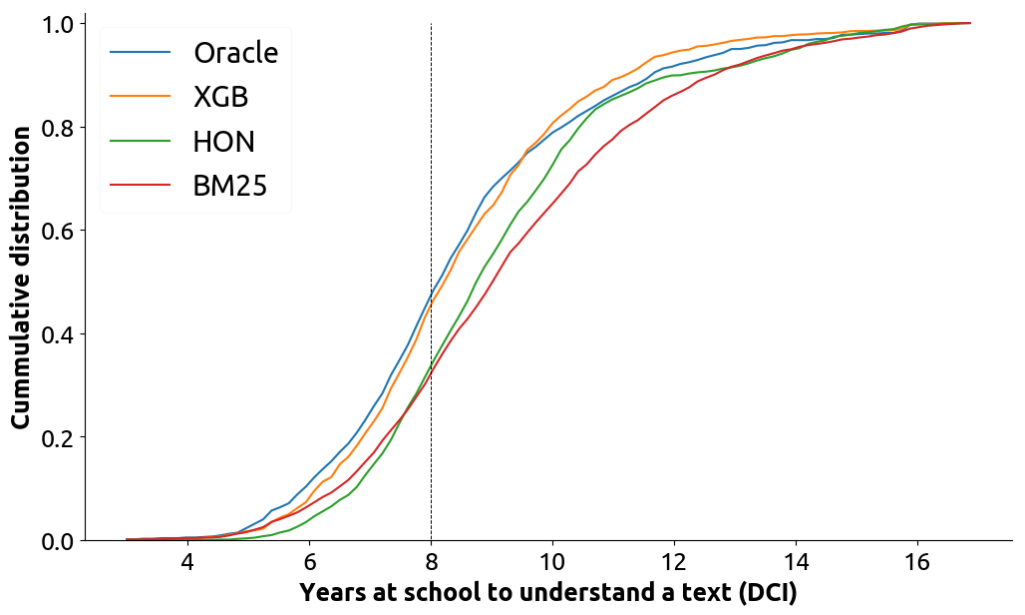
Cumulative distribution of Dale-Chall Index (DCI) of search results. DCI measures the years of schooling required to understand a document. The dashed line is the 8th grade level which is the reading level of an average US resident. The distribution for HealthOnNet (HON) is similar to that of the baseline used in this paper (Best Match 25 [BM25]). Our best method (eXtreme Gradient Boosting [XGB]) reranks documents to provide more understandable results; its distribution is similar to that of an oracle system.

As an illustrative example, we analyzed the top 10 search results retrieved by HON on October 01, 2017 in answer to 300 health search queries generated by regular health consumers in health forums. These queries are part of the Conference and Labs of the Evaluation Forum (CLEF) 2016 electronic health (eHealth) collection [14], which is extensively used in this paper. The understandability score of the retrieved pages was estimated with the most effective readability formula (RF) and preprocessing settings analyzed in this paper (low scores correspond to easy to understand Web pages). Figure 1 reports the cumulative distribution of understandability scores for these search results (note, we did not assess their topical relevance here). Dale-Chall Index (DCI) measures the years of schooling required to understand a document. The average US resident reads at or below an 8th grade level [15-18], which is the level suggested by the American National Institutes of Health for health information on the Web [19]. We also report the scores for the *optimal* search results (Oracle), as found in CLEF 2016 (relevant results that have the highest understandability scores), along with the scores for the baseline method (Best Match 25 [BM25]) and our best retrieval method, eXtreme Gradient Boosting (XGB). The results clearly indicate that despite solutions such as HON being explicitly aimed at supporting access to high-quality health information that can aid the user to take well-informed health decisions, they often fail to direct the users to information they can understand.

In this paper, we aim to establish methods and best practice for developing search engines that retrieve *relevant and understandable* health advice from the Web. The overall contributions of this paper can be summarized as:

1. We propose and investigate methods for the estimation of the understandability of health information in Web pages: a large number of medically focused features are grouped in categories and their contribution to the understandability estimation task is carefully measured.
2. We further study the influence of HTML processing methods on these estimations and their pitfalls, extending our previous work that has shown how this often-ignored aspect greatly impacts effectiveness [20].
3. We further investigate how understandability estimations can be integrated into retrieval methods to enhance the quality of the retrieved health information, with particular attention to its understandability by the general public. New models are explored in this paper, also extending our previous work [21].

This paper makes concrete contributions to practice, as it informs health search engines specifically tailored to the general public (eg, the HON or HealthDirect services referred to above) about the best methods they should adopt. These are novel and significant contributions as no previous work has systematically analyzed the influence of the components in this study—we show that these greatly influence retrieval effectiveness and, thus, delivery of relevant and understandable health advice.

### Related Work

Understandability refers to the ease of comprehension of the information presented to a user. In other words, health information is understandable “when consumers of diverse backgrounds and varying levels of health literacy can process and explain key messages” [22]. Often the terms understandability and readability are used interchangeably: we use readability to refer to formulae that estimate how easy it is to understand a text, usually based on its words and sentences. We use understandability to refer to the broader concept of ease of understanding: this is affected by text readability (as increasing readability tends to improve understanding) but might also be influenced by how legible a text is and its layout, including, for example, the use of images to explain difficult concepts.

There is a large body of literature that has examined the understandability of Web health content when the information seeker is a member of the general public. For example, Becker reported that the majority of health websites are not well designed for the elderly [23], whereas Stossel et al found that health education material on the Web is not written at an adequate reading level [18]. Zheng and Yu have reported on the readability of electronic health records compared with Wikipedia pages related to diabetes and found that readability measures often do not align with user ratings of readability [24]. A common finding of these studies is that, in general, health content available on Web pages is often hard to understand by the general public; this includes content that is retrieved in top-ranked positions by current commercial search engines [6-11].

Previous linguistics and information retrieval research has attempted to devise computational methods for the automatic estimation of text readability and understandability, and for the inclusion of these within search methods or their evaluation. Computational approaches to understandability estimations include (1) *RF*, which generally exploit word surface characteristics of the text, (2) *machine learning* approaches, and (3) matching with specialized *dictionaries or terminologies*, often compiled with information about understandability difficulty.

Measures such as Coleman-Liau Index (CLI) [25], DCI [26], and Flesch Reading Ease (FRE) [27] belong to the first category. These measures generally rely on surface-level characteristics of text such as characters, syllables, and word counts [28]. Although these measures have been widely used in studies investigating the understandability of health content retrieved by search engines [6-11,18,23]), our preliminary work found that these measures are heavily affected by the methods used to extract text from the HTML source [20]. We were able to identify specific settings of an HTML preprocessing pipeline that provided consistent estimates, but because of the lack of human assessments, we were not able to investigate how well each HTML preprocessing pipeline correlated with human assessments. In this paper, we revisited and extended this work in more detail, as we further investigated this problem by comparing the effect of HTML preprocessing on text understandability estimations in light of explicit human assessments.

The use of machine learning to estimate understandability forms an alternative approach. Earlier research explored the use of statistical natural language processing and language modeling [29-31] as well as linguistic factors such as syntactic features or lexical cohesion [32]. Although we replicated here many of the features devised in these works, they focus on estimating readability of general English documents rather than medical ones. In the medical domain, Zeng et al explored features such as word frequency in different medical corpora to estimate concept familiarity, which prompted the construction of the consumer health vocabulary (CHV) [33-35].

The actual use of CHV or other terminologies such as the Medical Subject Headings (MeSH) belongs to the third category of approaches. The CHV is a prominent medical vocabulary dedicated to mapping layperson vocabulary to technical terms [34]. It attributes a score for each of its concepts with respect to their difficulty, with lower or higher scores for harder or easier concepts. Researchers have evaluated CHV in tasks such as document analysis [36] and medical expertise prediction [37]. The hierarchy of MeSH was previously used in the literature to identify difficult concepts, assuming that a concept deep in the hierarchy is more difficult than a shallow one [38]. Other approaches combined vocabularies with word surface characteristics and syntactic features, such as part of speech (POS), into a unique readability measure [39].

In this study, we investigated approaches to estimate understandability from each of these categories, including measure the influence of HTML preprocessing on automatic understandability methods and establish best practices.

Some previous works have attempted to use understandability estimations for improving search results in consumer health search as well as methods to evaluate retrieval systems that do account for understandability along with topical relevance. Palotti et al have used learning to rank with standard retrieval features along with features based on RF and medical lexical aspects to determine understandability [21]. Van Doorn et al have shown that learning a set of rankers that provide trade-offs across a number of relevance criteria, including readability or understandability, increases overall system effectiveness [40]. Zuccon and Koopman [41], and later Zuccon [42], have proposed and investigated a family of measures based on the gain-discount framework, where the gain of a document is influenced by both its topical relevance and its understandability. They showed that although generally correlated, topical relevance evaluation alone provides differing system rankings compared with understandability-biased evaluation measures. In this study, we further explored the development of retrieval methods that combine signals about topical relevance and understandability.

## Methods

### Data Collection

In this paper, we investigated methods to estimate Web page understandability, including the effect that HTML preprocessing pipelines and heuristics have, and their search effectiveness when employed within retrieval methods. To obtain both topical relevance and understandability assessments, we used the data from the CLEF 2015 and 2016 eHealth collections. The CLEF eHealth initiative is a research community–shared task aimed at creating resources for evaluating health search engines aimed at the general public [43]. Note, in the remainder of this paper, we refer to topical relevance simply as relevance, when this does not cause confusion.

The CLEF 2015 collection contains 50 queries and 1437 documents that have been assessed as relevant by clinical experts and have an assessment for understandability [44]. Documents in this collection are a selected crawl of health websites, of which the majority are certified HON websites. The CLEF 2016 collection contains 300 queries and 3298 relevant documents that also have been assessed with respect to understandability [14]. Documents in this collection belong to the ClueWeb12 B13 corpus [45], and thus are general English Web pages, not necessarily targeted to health topics nor of a controlled quality (as are the HON certified pages). Understandability assessments were provided on a 5-point Likert scale for CLEF 2015 on a 0 to 100 range for CLEF 2016 (0 indicates the highest understandability).

To support the investigation of methods to automatically estimate the understandability of Web pages, we further considered correlations between multiple human assessors (interassessor agreement). For CLEF 2015, we used the publicly available additional assessments made by unpaid medical students and health consumers collected by Palotti et al [46] in a study of how medical expertise affects assessments. For CLEF 2016, we collected understandability assessments for 100 documents. In total, 3 members of our research team, who did not author this paper and are not medical experts, were recruited to provide the assessments (the correlation of these additional assessments and CLEF’s ground truth is examined further in this paper). The Relevation tool [47] was used to assist with the assessments, mimicking the settings used in CLEF.

### Understandability Estimators

Several methods have been used to estimate the understandability of health Web pages, with the most popular methods (at least in the biomedical literature) being RF based on surface level characteristics of the text. Next, we outline the categories of methods to estimate understandability used in this study; an overview is shown in Textboxes 1 to 10.

#### Traditional Readability Formulae

These include the most popular RF [25-27] as well as other less popular ones [48-51]. An extensive description of these RF is provided in surveys by Collins-Thompson [52] and Dubay [28]. A complete list of methods is provided in Textbox 1.

#### Raw Components of Readability Formulae

These are formed by the *building blocks* used in the traditional RF. Examples include the average number of characters per word and the average number of syllables in a sentence. Words are divided into syllables using the Python package Pyphen [53]. A complete list of methods is provided in Textbox 2.

#### General Medical Vocabularies

These include methods that count the number of words with a medical prefix or suffix, that is, beginning or ending with Latin or Greek particles (eg, amni-, angi-, algia-, and arteri-), and text strings included in lists of acronyms or in medical vocabularies such as the International Statistical Classification of Diseases and Related Health Problems (ICD), Drugbank and the OpenMedSpel dictionary [54]. An acronym list from the ADAM database [55] was used. Methods in this category were matched with documents using simple keyword matching. A complete list of methods is provided in Textbox 3.

#### Consumer Medical Vocabulary

The popular MetaMap [56] tool was used to map the text content of Web pages to entries in CHV [34]. We used the MetaMap semantic types to retain only concepts identified as symptoms or diseases. Similar approaches have been commonly used in the literature [57-60]. A complete list of methods is provided in Textbox 4.

#### Expert Medical Vocabulary

Similar to the CHV features, we used MetaMap to convert the content of Web pages into MeSH entities, studying symptom and disease concepts separately. A complete list of methods is provided in Textbox 5.

#### Natural Language Features

These included commonly used natural language heuristics such as the ratio of POS classes, the height of the POS parser tree, the number of entities in the text, the sentiment polarity [61], and the ratio of words found in English vocabularies. The Python package Natural Language Toolkit [62] was used for sentiment analysis, POS tagging, and entity recognition. The GNU Aspell [63] dictionary was used as a standard English vocabulary and a stop word list was built by merging those of Indri [64] and Terrier [65]. Discourse features, such as the distribution of POS classes and density of entity in a text, were previously studied in the task of understandability prediction [66] and found superior to complex features such as entity coreference and entity grid [67]. To the best of our knowledge, sentiment polarity was never investigated in this task. Our intuition is that the content produced by laypeople in patient forums or blogs (easy to read) is potentially more emotional than scientific publications (hard to read). A complete list of methods is provided in Textbox 6.

#### HTML Features

These include the identification of a large number of HTML tags, which were extracted with the Python library BeautifulSoup [68]. The intuition for these features is that Web pages with many images and tables might explain and summarize health content better, thus providing more understandable content to the general public. A complete list of methods is provided in Textbox 7.

**Readability formulae (RF) used to estimate understandability.**

Readability feature

- Automated Readability Index [48]
- Coleman-Liau Index (CLI) [25]
- Dale-Chall Index (DCI) [26]
- Flesch-Kincaid Grade Level [27]
- Flesch Reading Ease (FRE) [27]
- Gunning Fog Index (GFI) [49]
- Lasbarhetsindex (LIX) [50]
- Simple Measure of Gobbledygook (SMOG) [51]

**Raw components of readability formulae (CRF) used to estimate understandability. For all features, raw values, values normalized by number of words in a document, and values normalized by number of sentences in a document were used.**

Components of readability feature

- # of Characters
- # of Words
- # of Sentences
- # of Difficult Words (Dale-Chall list [26])
- # of Words Longer than 4 Characters
- # of Words Longer than 6 Characters
- # of Words Longer than 10 Characters
- # of Words Longer than 13 Characters
- # of Number of Syllables
- # of Polysyllable Words (>3 Syllables)

**General medical vocabulary features used to estimate understandability. For all features, raw values, values normalized by number of words in a document, and values normalized by number of sentences in a document were used.**

General medical vocabularies (GMVs)

- # of words with medical prefix
- # of words with medical suffix
- # of acronyms
- # of International Statistical Classification of Diseases and Related Health Problems (ICD) concepts
- # of Drugbank
- # of words in medical dictionary (OpenMedSpel)

**Consumer medical vocabulary features used to estimate understandability. For all features, raw values, values normalized by number of words in a document, and values normalized by number of sentences in a document were used.**

Consumer medical vocabularies (CMV)

- Consumer health vocabulary (CHV) mean score for all concepts
- # of CHV concepts
- CHV mean score for symptom concepts
- # of CHV symptom concepts
- CHV mean score for disease concepts
- # of CHV disease concepts

**Expert medical vocabulary features used to estimate understandability. For all features, raw values, values normalized by number of words in a document, and values normalized by number of sentences in a document were used.**

Expert medical vocabulary (EMV)

- # of Medical Subject Headings (MeSH) concepts
- Average tree of MeSH concepts
- # of MeSH symptom concepts
- Average tree of MeSH symptom concepts
- # of MeSH disease concepts
- Average tree of MeSH disease concepts

**Natural language features used to estimate understandability. For all features, raw values, values normalized by number of words in a document, and values normalized by number of sentences in a document were used.**

Natural language features (NLF)

- Positive words
- Negative words
- Neutral words
- # of verbs
- # of nouns
- # of pronouns
- # of adjectives
- # of adverbs
- # of adpositions
- # of conjunctions
- # of determiners
- # of cardinal numbers
- # of particles or other function words
- # of other part of speech (POS; foreign words and typos)
- # of punctuation
- # of entities
- Height of POS parser tree
- # of stop words
- # of words not found in Aspell Engish dictionary
- Average tree of Medical Subject Headings (MeSH) disease concepts

#### Word Frequency Features

Generally speaking, common and known words are usually frequent words, whereas unknown and obscure words are generally rare. This idea is implemented in RF such as the DCI, which uses a list of common words and counts the number of words that fall outside this list (complex words) [26] and has shown success in other recent approaches [69,70]. We extended these observations by studying corpus-wide word frequencies. In total, 3 corpora were analyzed to extract word frequencies:

- Medical Reddit: Reddit [71] is a Web forum with a sizeable user community, which is responsible for generating and moderating its content. This forum is intensively used for health purposes, for example, in the Reddit community AskDocs [72], licensed nurses and doctors (subject to user identity verification) advise help seekers free of charge. We selected 6 of such communities (medical, AskDocs, AskDoctorSmeeee, Health, WomensHealth, and Mens_Health) and downloaded all user interactions available until September 1, 2017, using the Python library Python Reddit Wrapper PRAW [73]. In total, 43,019 discussions were collected.
- Medical English Wikipedia: after obtaining a recent Wikipedia dump [74] (May 1, 2017), we filtered papers to only those containing an Infobox in which at least one of the following words appeared as a property: ICD10, ICD9, DiseasesDB, MeSH, MeSHID, MeshName, MeshNumber, GeneReviewsName, Orphanet, eMedicine, MedlinePlus, drug_name, Drugs.com, DailyMedID, and LOINC. A Wikipedia infobox is a structured template that appears on the right of Wikipedia pages summarizing key aspects of papers. This process followed the method by Soldaini et al [75], which favors precision over recall when identifying a health-related paper. This resulted in a collection of 11,868 papers.
- PubMed Central: PubMed Central is a Web-based database of biomedical literature. We used the collection distributed for the Text Retrieval Conference (TREC) 2014 and 2015 Clinical Decision Support Track [76,77], consisting of 733,191 papers.

**HTML features used to estimate understandability.**

HTML features (HF)

- # of abbreviation (abbr tags)
- # of links (A tags)
- # of blockquote tags
- # of bold tags
- # of cite tags
- # of divisions or sections (div tags)
- # of forms tags
- # of heading H1 tags
- # of heading H2 tags
- # of heading H3 tags
- # of heading H4 tags
- # of heading H5 tags
- # of heading H6 tags
- Total # of headings (any heading H above)
- # of image tags
- # of input tags
- # of link tags
- # of description lists (DL tags)
- # of unordered lists (UL tags)
- # of ordered lists (OL tags)
- Total # of any list (DL+UL+OL)
- # of short quotations (Q tags)
- # of scripts tags
- # of spans tags
- # of table tags
- # of paragraphs (P tags)

A summary of the statistics of the corpora is reported in Table 1. We modeled word frequencies in a corpus in a straightforward manner: we sorted the word frequencies and normalized word rankings such that values close to 100 are attributed to common words and values close to 0 to rare words. Thereafter, we replaced each word in a document by a number ranging from 0 to 100, which represents the frequency of that word in the corpus. Finally, we extracted features based on the word frequency distribution for that document. For example, the feature *75th percentile English Wikipedia* is a number between 0 and 100 representing how frequent is the word at the 75th percentile of a document in which word frequencies were extracted from the English Wikipedia corpus. Unless explicitly stated otherwise, we ignored out-of-vocabulary (OV) words in the corpus. A complete list of methods is provided in Textbox 8.

#### Machine Learning on Text-Regressors and Classifiers

These include machine learning methods for estimating Web page understandability. Although Collins-Thompson highlighted the promise of estimating understandability using machine learning methods, a challenge is identifying the background corpus to be used for training [52]. To this aim, we used the 3 corpora detailed above, and assumed understandability labels according to the expected difficulty of documents in these collections:

- Medical Reddit (label 1): Documents in this corpus are expected to be written in a colloquial style, and thus the easiest to understand. All the conversations are, in fact, explicitly directed to assist inexpert health consumers
- Medical English Wikipedia (label 2): Documents in this corpus are expected to be less formal than scientific papers, but more formal than a Web forum like Reddit, thus somewhat more difficult to understand
- PubMed Central (label 3): Documents in this corpus are expected to be written in a highly formal style, as the target audience are physicians and biomedical researchers.

**Table 1:** Statistics for the corpora used as background models for understandability estimations.

Statistics	Medical Wikipedia	Medical Reddit	PubMed Central
Documents, n	11,868	43,019	733,191
Words, n	10,655,572	11,978,447	144,024,976
Unique words, n	467,650	317,106	2,933,167
Average words per document, mean (SD)	898.90 (1351.76)	278.45 (359.70)	227.22 (270.44)
Average characters per document, mean (SD)	5107.81(7618.57)	1258.44 (1659.96)	1309.11(1447.31)
Average characters per word, mean (SD)	5.68 (3.75)	4.52 (3.52)	5.76 (3.51)

**Word frequency features used to estimate understandability.**

Word frequency features (WFF)

- 25th percentile English Wikipedia
- 50th percentile English Wikipedia
- 75th percentile English Wikipedia
- Mean rank English Wikipedia
- Mean rank English Wikipedia—includes out-of-vocabulary (OV) words
- 25th percentile Medical Reddit
- 50th percentile Medical Reddit
- 75th percentile Medical Reddit
- Mean rank Medical Reddit
- Mean rank Medical Reddit—includes OV
- 25th percentile Pubmed
- 50th percentile Pubmed
- 75th percentile Pubmed
- Mean rank Pubmed
- Mean rank Pubmed—includes OV
- 25th percentile Wikipedia+Reddit+Pubmed
- 50th percentile Wikipedia+Reddit+Pubmed
- 75th percentile Wikipedia+Reddit+Pubmed
- Mean rank Wikipedia+Reddit+Pubmed
- Mean rank Wikipedia+Reddit+Pubmed—includes OV

**Machine learning regressor features used to estimate understandability.**

Machine learning regressors (MLR)

- Linear regressor
- Multilayer perceptron regressor
- Random forest regressor
- Support vector machine regressor
- eXtreme Gradient Boosting Regressor

**Machine learning classifier features used to estimate understandability.**

Machine learning classifiers (MLC)

- Logistic regression
- Multilayer perceptron classifier
- Random forest classifier
- Support vector machine classifier
- Multinomial naive Bayes
- eXtreme Gradient Boosting Classifier

On the basis of the labels of each class above, models were learnt using all documents from these corpora after features were extracted using latent semantic analysis with ten dimensions on top of TF-IDF calculated for each word. We modeled a classification task as well as a regression task using these corpora. In the classification task, the first step is to train a classifier on documents belonging to these three collections with the three different classes shown above. The second step is to use the classifier to estimate which of these three possible classes an unseen document from the CLEF 2015 or CLEF 2016 would belong. Similarly, in the regression task, after training, a regressor has to estimate an understandability value to an unseen CLEF document. We hypothesize that documents that are more difficult to read are more similar to PubMed documents than to Wikipedia or Reddit ones. A complete list of methods is provided in Textboxes 9 and 10.

### Preprocessing Pipelines and Heuristics

As part of our study, we investigated the influence that the preprocessing of Web pages had on the estimation of understandability computed using the methods described above. We did so by comparing the combination of a number of preprocessing pipelines, heuristics, and understandability estimation methods with human assessments of Web page understandability. Our experiments extended our previous work [20] and provided a much more thorough analysis, as they only evaluated surface level RF and did not compare their results against human assessments.

To extract the content of a Web page from the HTML source we tested: BeautifulSoup, *Naive* [68], which just naively removes HTML tags and Boilerpipe, *Boi* [78] and Justext, *Jst* [79], which eliminates boilerplate text together with HTML tags. Our data analysis in Palotti et al [20] highlighted that the text in HTML fields such as titles, menus, tables, and lists often missed a correct punctuation mark, and thus, the text extracted from them could be interpreted as many short sentences or few very long sentences, depending on whether a period was forced at the end of fields or sentences. We, thus, implemented the same 2 heuristics devised to deal with this: *ForcePeriod (FP)* and *DoNotForcePeriod (DNFP)*. If a punctuation mark is found at the end of a field or sentence, it is kept as it is. However, if no punctuation mark is found at the end of a field or sentence, the FP heuristic forces the insertion of a period at the end of that extracted HTML field, whereas the DNFP does not.

### Integrating Understandability into Retrieval

We then investigated how understandability estimations can be integrated into retrieval methods to increase the quality of search results. Specifically, we considered 3 retrieval methods of differing quality for the initial retrieval. These included the best 2 runs submitted to each CLEF task, and a plain BM25 baseline (default Terrier parameters: b=0.75 and k_1_=1.2). BM25 is a probabilistic term weighting scheme commonly used in information retrieval and is defined with respect to the frequency of a term in a document, the collection frequency of that term, and the ratio between the length of the document and the average document length. As understandability estimators, we used the XGB regressor [80] as well as Simple Measure of Gobbledygook (SMOG) for CLEF 2015 and DCI for CLEF 2016. These were selected as they were the best performing RF and machine learning methods for each collection (details on the evaluation of understandability estimators presented in the Results section). Remember that, as described in the *Related Work* section, RF are a specific approach to estimate understandability. Note that in XGB, for assessed documents we used 10-fold cross validation, training XGB on 90% of the data, and used its predictions for the remaining 10%. For unassessed documents, we trained XGB on all assessed data and applied this model to generate predictions. Different machine learning methods and feature selection schemes were experimented with; results are available in the Multimedia Appendix 1. XGB was selected because its results were the best among the machine learning test (which include all machine learning methods listed in Textboxes 9 and 10).

To integrate understandability estimators into the retrieval process, we first investigated *reranking* search results retrieved by the initial runs purely based on the understandability estimations. If all the search results from a run were to be considered, then such a reranking method might place at early ranks Web pages highly likely to be understandable, but possibly less likely to be topically relevant. To balance relevance and understandability, we only reranked the first *k* documents. We explored rank cut-offs k=15,20,50. As evaluation was performed with respect to the first n=10 rank positions, the setting k=15 provided a conservative reranking of search results, whereas, k=50 provided a less conservative reranking approach.

**Table 2:** Learning to rank settings.

Strategy	Explanation	Labeling function	
		CLEF^a^ 2015	CLEF 2016
LTR^b^ 1	Model built *only* on the topicality labels with IR^c^ features	F^d^(R^e^,U^f^)=R	F(R,U)=R
LTR 2	Model built *only* on the topicality labels with IR and understandability features	F(R,U)=R	F(R,U)=R
LTR 3	Model combines understandability and topicality labels. Uses IR and understandability features	F(R,U)=R×U/3	F(R,U)=R×(100-U)/100
LTR 4	Model built *only* on easy-to-read documents. Uses IR and understandability Features	F(R,U)=R, if U≥2 F(R,U)=0, otherwise	F(R,U)=R, if U≤40 F(R,U)=0, otherwise
LTR 5	Model built boosting easy-to-read documents. Uses IR and understandability Features	F(R,U)=2×R, if U≥2 F(R,U)=R, otherwise	F(R,U)=2×R, if U≤40 F(R,U)=R, otherwise

^a^CLEF: Conference and Labs of the Evaluation Forum.

^b^LTR: learning to rank.

^c^IR: information retrieval.

^d^F: function.

^e^R: topical relevance of a document.

^f^U: understandability.

As an alternative to the previous 2-step ranking strategy for combining topical relevance and understandability, we explored the *fusion* of 2 search result lists separately obtained for relevance and understandability. For this, we used the reciprocal rank fusion method [81], which was shown effective for combining 2 lists of search results based on their documents’ *ranks*, rather than scores. This approach was selected above score-based fusion methods because the distribution of relevance scores for the retrieved documents differed sensibly (both in magnitude and spread) with that of understandability scores: in such a case, score-based fusion is not appropriate. For relevance, we used, separately, the 3 retrieval methods for each collection. For CLEF 2015, we used BM25 and the submissions made by the East China Normal University (ECNU) team [82] and the Korean Institute of Science and Technology Information (KISTI) team [83]. For CLEF2016, we also used BM25 and the submissions made by the Georgetown University Information Retrieval (GUIR) team [84] and ECNU [85]. For understandability, we used, separately, the estimations from SMOG or DCI and XGB. Moreover, for this approach, we studied limiting the ranking of results to be considered by the methods across the cut-offs k=15,20,50.

Finally, we considered a third alternative to combine relevance and understandability: using *learning to rank* with features derived from retrieval methods (information retrieval (IR) features) and understandability estimators. Learning to rank refers to a family of machine learning methods where ranking models are learnt from training data (and associated features). With the CLEF 2015 and 2016 collections, we explored 5 combinations of label attribution and feature sets, maintaining the same pairwise learning to rank algorithm based on tree boosting (XGB). These combinations are listed in Table 2, with R being the relevance of documents and U their understandability estimation. Although the definitions of learning to rank (LTR) 1 and LTR 2 are straightforward, the other methods deserve some further explanation. In LTR 3, a penalty was proportionally assigned to documents according to their understandability score U. For example, for CLEF 2016, a document with understandability U=0 received no penalty, as 0 was the easiest level of understanding, whereas another with understandability 50 received a 50% penalty, meaning that its relevance score was halved. LTRs 4 and 5 were based on a fixed threshold applied to the understandability score: if the score was higher than the threshold (U=2 for CLEF 2015 and U=40 for CLEF 2016), then the original relevance score (for LTR 4) or a boosted value (for LTR 5) was assigned to the corresponding document. We used the thresholds U=2 for CLEF 2015 and U=40 for CLEF 2016, based on the distribution of understandability assessments and the semantic of understandability labels [44,14].

### Evaluation Measures

In the experiments, we used Pearson, Kendall, and Spearman correlations to compare the understandability assessments of human assessors with estimations obtained by the considered automated approaches, under all combinations of pipelines and heuristics. Pearson correlation is used to calculate the strength of the linear relationship between 2 variables, whereas Kendall and Spearman measure the rank correlations among the variables. We opted to report all 3 correlation coefficients to allow for a thorough comparison with other work, as they are equally used in the literature.

For the retrieval experiments, we used evaluation measures that rely on both (topical) relevance and understandability. The uRBP measure [42] extends rank biased precision (RBP) to situations where multiple relevance dimensions are used. The measure is formulated as uRBP(p)=(1-p) × ∑^k^ p^k-1^ × r(d@k) × u(d@k), where r(d@k) is the gain for retrieving a relevant document at rank k and u(d@k) is the gain for retrieving a document of a certain understandability at rank k; p is the RBP persistence parameter. This measure was an official evaluation measure used in CLEF (we also set *P*=.8).

A drawback of uRBP is that relevance and understandability are combined into a unique evaluation score, thus making it difficult to interpret whether improvements are because of more understandable or more topical documents being retrieved. To overcome this, we used the multidimensional metric (MM) framework introduced by Palotti et al [86] which first separately calculates an RBP value for relevance and another for understandability, and then combine them into a unique effectiveness measure:

- *RBP*_*r*_*@n(p)*: uses the relevance assessments for the top *n* search results (ie, this is the common RBP). We regarded a document as topically relevant if assessed as somewhat relevant or highly relevant.
- *RBP*_*u*_*@n(p)*: uses the understandability assessments for the top *n* search results. We regarded a document as understandable (1) for CLEF 2015 if assessed easy or somewhat easy to understand and (2) for CLEF 2016 if its assessed understandability score was smaller than a threshold U. We used U=40, based on the distribution of understandability assessments. Assessors were presented with a slider for understandability assessment and U=50 was labeled as average understandability. This created a bimodal distribution of understandability assessments, with U=40 being a good upper limit for easy-to-read documents. The understandability distribution can be found in the Multimedia Appendix 2.

- *MM*_*RBP*_*@n(p)=2×(RBP*_*r*_*@n×RBP*_*u*_*@n)/(RBP*_*r*_*@n+RBP*_*u*_*@n)*: combines the previous 2 RBP values into a unique measurement using the harmonic mean (in the same fashion that the F_1_ measure combines recall and precision).

For all measures, we set n=10 because shallow pools were used in CLEF along with measures that focused on the top 10 search results (including RBP_r_@10). Shallow pools refer to the selection of a limited number of documents to be assessed for relevance, among the documents retrieved at the top ranks by a search engine.

Along with these measures of search effectiveness, we also recorded the number of unassessed documents, the RBP residuals, RBP^*^_r_@10, RBP^*^_u_@10, and MM^*^_RBP_, that is, the corresponding measures calculated by ignoring unassessed documents. These latter measures implement the condensed measures approach proposed by Sakai as a way to deal with unassessed documents [87]. We did this to minimize pool bias as the pools built in CLEF were of limited size and the investigated methods retrieved a substantial number of unassessed documents. Pool bias refers to the possible bias in the evaluation toward systems that have contributed documents to the assessment pool: these erroneously receive higher evaluation scores compared with systems that did not contribute to the pool (ie, that were not sampled to create the set of documents to be judged for relevance).

## Results

### Evaluation of Understandability Estimators

To keep this paper succinct, in the following we only report a subset of the results. The remaining results (which show similar trends to those reported here) are made available in the Multimedia Appendix 3 material for completeness.

Using the CLEF eHealth 2015 and 2016 collections, we studied the correlations of methods to estimate Web page understandability compared with human assessments. For each category of understandability estimation, Tables 3 and 4 report the methods with highest Pearson and Spearman or Kendall correlations for CLEF 2015 and 2016, respectively. For each method, we used the best preprocessing settings; a study of the impact of preprocessing is reported in the next subsection.

Overall, Spearman and Kendall correlations obtained similar results (in terms of which methods exhibited the highest correlations): this was expected as, unlike Pearson, they are both rank-based correlations.

For traditional RF, SMOG had the highest correlations for CLEF 2015 and DCI for CLEF 2016, regardless of correlation measure. These results resonate with those obtained for the category of raw components of readability formulae (CRF). In fact, the polysyllable words measure, which is the main feature used in SMOG, had the highest correlation for CLEF 2015 among methods in this category. Similarly, the number of difficult words, which is the main feature used in DCI, had the highest correlation for CLEF 2016 among methods in this category.

When examining the expert vocabulary category (EMV), we found that the number of MeSH concepts obtained the highest correlations with human assessments; however, its correlations were substantially lower than those achieved by the best method from the consumer medical vocabularies category (CMV), that is, the scores of CHV concepts. For the natural language category (NLF), we found that the number of pronouns, the number of stop words, and the number of OV words had the highest correlations—and these were even higher than those obtained with MeSH- and CHV-based methods. In turn, the methods that obtained the highest correlations among the HTML category (HF) and counts of P tags and list tags exhibited overall the lowest correlations compared with methods in the other categories. P tags are used to create paragraphs in a Web page, being thus a rough proxy for text length. Among methods in the word frequency category (WFF), the use of Medical Reddit (but also of PubMed) showed the highest correlations, and these were comparable with those obtained by the RF.

Finally, regressors (MLR) and classifiers (MLC) exhibited the highest correlations among all methods: in this category, the XGB regressor and the multinomial Naive Bayes best correlated with human assessments.

**Table 3:** Methods with the highest correlation per category for Conference and Labs of the Evaluation Forum (CLEF) 2015.

Category	Method	Preprocessing	Pearson	Spearman	Kendall
Readability formulae	Simple Measure of Gobbledygook Index	Jst Do Not Force Period (DNFP)	*.438* ^a^	*.388*	*.286*
Components of readability formulae (CRF)	Average number of Polysyllables words per sentence	Jst force period (FP)	*.429*	.364	.268
CRF	Average number of Polysyllables words per sentence	Jst DNFP	.192	*.388*	*.286*
General medical vocabularies (GMVs)	Average number of medical prefixes per word	Naïve FP	*.314*	.312	.229
GMVs	Number of medical prefixes	Naïve FP	.131	*.368*	*.272*
Consumer medical vocabulary (CMV)	Consumer health vocabulary (CHV) mean score for all concepts	Naïve FP	*.371*	*.314*	*.228*
Expert medical vocabulary (EMV)	Number of medical concepts	Naïve FP	*.227*	*.249*	*.178*
Natural language features (NLF)	Number of words not found in Aspell dictionary	Jst DNFP	*.351*	.276	.203
NLF	Number of pronouns per word	Naïve FP	.271	*.441*	*.325*
HTML features (HF)	Number of P tags	None	*.219*	*.196*	*.142*
Word frequency features (WFF)	Mean rank Medical Reddit	Jst DNFP	*.435*	.277	.197
WFF	25th percentile Pubmed	Jst DNFP	.330	*.347*	*.256*
Machine learning regressors (MLR)	eXtreme Gradient Boosting (XGB) Regressor	Boi DNFP	*.602*	.394	.287
MLR	XGB Regressor	Jst FP	.565	*.438*	*.324*
Machine learning classifiers	Multinomial Naïve Bayes	Naïve FP	*.573*	*.477*	*.416*

^a^Italics used to highlight the best result of each group.

**Table 4:** Methods with the highest correlation per category for Conference and Labs of the Evaluation Forum (CLEF) 2016.

Category	Method	Preprocessing	Pearson	Spearman	Kendall
Readability formulae (RF)	Dale-Chall Index (DCI)	Jst force period (FP)	*.439* ^a^	.381	*.264*
RF	DCI	Boi FP	.437	*.382*	*.264*
Components of readability formulae (CRF)	Average number of difficult word per Word	Boi FP	*.431*	*.379*	*.262*
General medical vocabularies (GMVs)	Average prefixes per sentence	Jst FP	*.263*	.242	.164
GMVs	International Statistical Classification of Diseases and Related Health Problems concepts per sentence	Jst do not force period (DNFP)	.014	*.253*	*.172*
Consumer medical vocabulary (CMV)	Consumer health vocabulary (CHV) mean score for all concepts	Jst FP	*.329*	.313	.216
CMV	CHV mean score for all concepts	Boi FP	*.329*	*.325*	*.224*
EMV	Number of MeSH (Medical Subject Headings) concepts	Boi DNFP	*.201*	.166	.113
Expert medical vocabulary (EMV)	Number of MeSH disease concepts	Boi DNFP	.179	*.192*	*.132*
Natural language features (NLF)	Average stop word per word	Boi FP	*.344*	.312	.213
NLF	Number of pronouns	Boi FP	.341	*.364*	*.252*
HTML features (HF)	Number of lists	None	*.114*	.021	.015
HF	Number of P tags	None	.110	*.123*	*.084*
Word frequency features (WFF)	Mean rank Medical Reddit	Boi DNFP	*.387*	.312	.214
WFF	50th percentile Medical Reddit	Jst DNFP	.351	*.315*	*.216*
Machine learning regressors (MLR)	eXtreme Gradient Boosting (XGB) Regressor	Jst DNFP	*.454*	*.373*	.258
MLR	Random Forest Regressor	Boi DNFP	.389	.355	*.264*
Machine learning classifiers	Multinomial Naïve Bayes	Jst FP	*.461*	*.391*	*.318*

^a^Italics used to highlight the best result of each group.

### Evaluation of Preprocessing Pipelines and Heuristics

**Figure 2:**
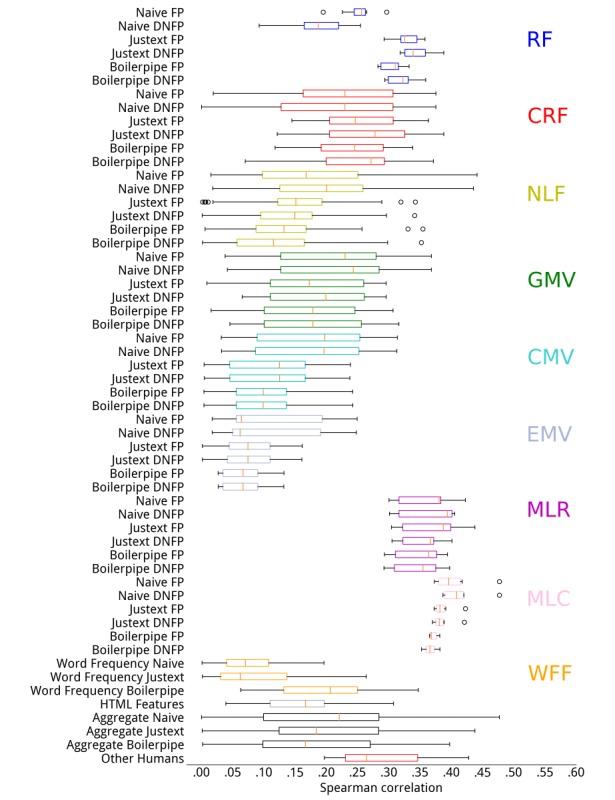
Correlations between understandability estimators and human assessments for Conference and Labs of the Evaluation Forum 2015. For example, the first boxplot on the top represents the distribution of Spearman correlations with human assessments across all features in the category readability formulae, obtained with the Naive Force Period preprocessing. Each box extends from the lower to the upper quartile values, with the red marker representing the median value for that category. Whiskers show the range of the data in each category and circles represent values considered outliers for the category (eg, Spearman correlation for Simple Measure of Gobbledygook (SMOG) index was .296 and for Automated Readability Index (ARI) was .194: these were outliers for that category). CMV: consumer medical vocabulary; CRF: components of readability formulae; DNFP: Do Not Force Period; EMV: expert medical vocabulary; FP: Force Period; GMV: general medical vocabulary; MLC: machine learning classifiers; MLR: machine learning regressors; NLF: natural language features; RF: readability formulae; WFF: word frequency features.

**Figure 3:**
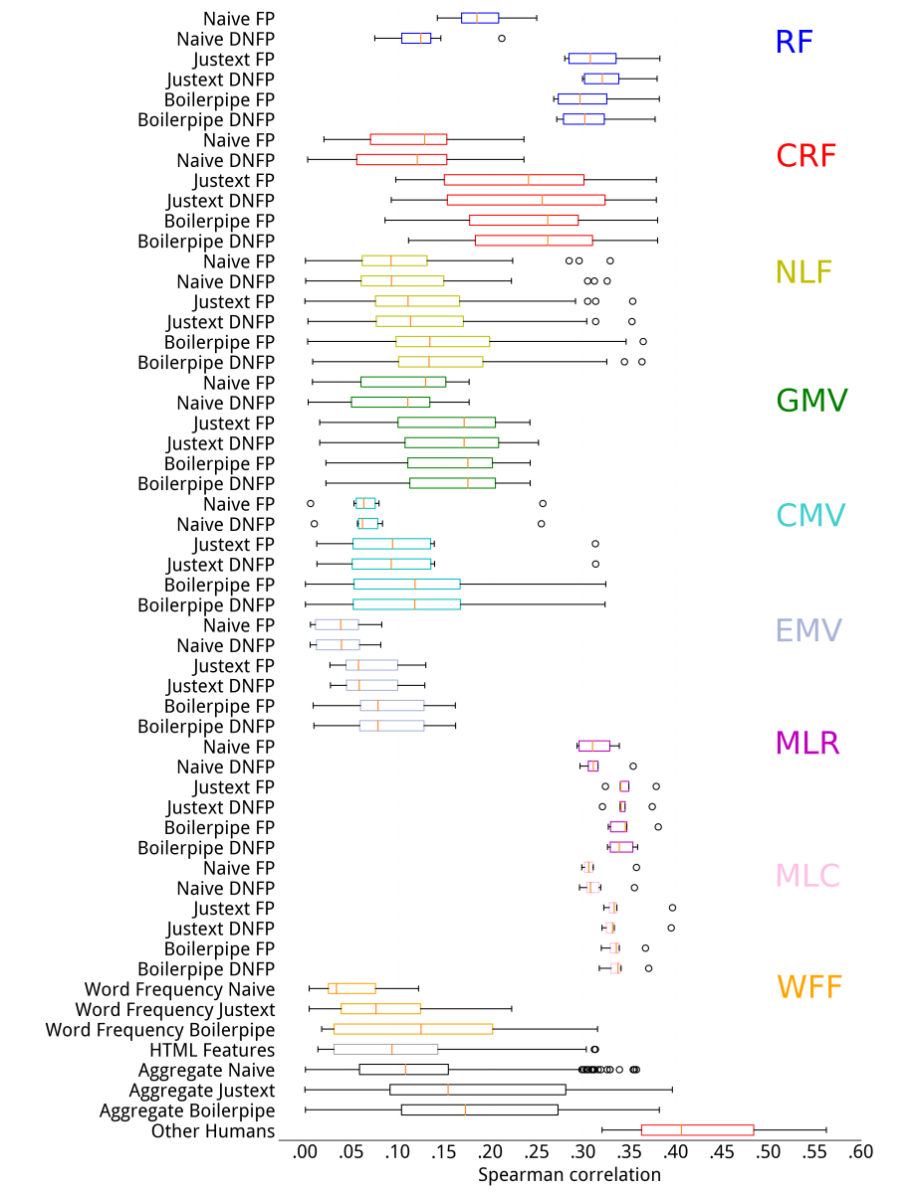
Correlations between understandability estimators and human assessments for Conference and Labs of the Evaluation Forum (CLEF) 2016. CMV: consumer medical vocabulary; CRF: components of readability formulae; DNFP: Do Not Force Period; EMV: expert medical vocabulary; FP: Force Period; GMV: general medical vocabulary; MLC: machine learning classifiers; MLR: machine learning regressors; NLF: natural language features; RF: readability formulae; WFF: word frequency features.

Results from experiments with different preprocessing pipelines and heuristics are shown in Figures 2 and 3, respectively for CLEF 2015 and 2016. For each category of methods and combination of preprocessing and heuristics, we report their variability in terms of Spearman rank correlation with human assessments. Results for Pearson and Kendall correlations are reported in the Multimedia Appendix 3, but showed similar trends. We further report the summary results across all understandability assessment methods and sentence-ending heuristics for each of the preprocessing pipelines. Finally, we also report the interassessor correlation (last box) when multiple assessors provided judgments about the understandability of Web pages. This provides an indication of the range of variability and subjectiveness when assessing understandability, along with the highest correlation we measured between human assessors.

We first examined the correlations between human assessments and RF. We found that the *Naive* preprocessing resulted in the lowest correlations, regardless of RF and heuristic (although *DoNotForcePeriod* performed better than *ForcePeriod*). Using Justext or Boilerplate resulted in higher correlations with human understandability assessments, and the *ForcePeriod* heuristic was shown to be better than *DoNotForcePeriod*. These results confirm our hypotheses in Palotti et al [20]: we found these settings to produce lower variances in understandability estimations, and thus hypothesized that they were better suited to the task.

Overall, among RF, the best results (highest correlations) were obtained by SMOG and DCI (see also Tables 3 and 4). Although no single setting outperformed the others in both collections, we found that the use of CLI and FRE with *Justext* provided the most stable results across the collections, with correlations as high as the best ones in both collections. These results confirmed our previous advice [20], that is, in general, if using readability measures, CLI is to be preferred, along with an appropriate HTML extraction pipeline, regardless of the heuristic for sentence ending. We provide detailed plots to compare the results in this paper with those in Palotti et al [20] in the Multimedia Appendix 4.

When considering methods beyond those based on RF, we found that the highest correlations were achieved by the regressors (MLR) and classifiers (MLC), independently of the preprocessing method used. There is little difference in terms of effectiveness of methods in these categories, with the exception of regressors on CLEF 2015 that exhibited not negligible variances: whereas for the neural network regressor the Pearson correlation was .44 and for the support vector regressor it was only .30.

A common trend when comparing preprocessing pipelines is that the Naïve pipeline provided the weakest correlations with human assessments for CLEF 2016, regardless of estimation methods and heuristics. This result, however, was not confirmed for CLEF 2015, where the Naive preprocessing negatively influenced correlations for the RF category, but not for other categories, although it was generally associated with larger variances for the correlation coefficients.

### Evaluation of Understandability Retrieval

#### Reranking Experiments

**Figure 4:**
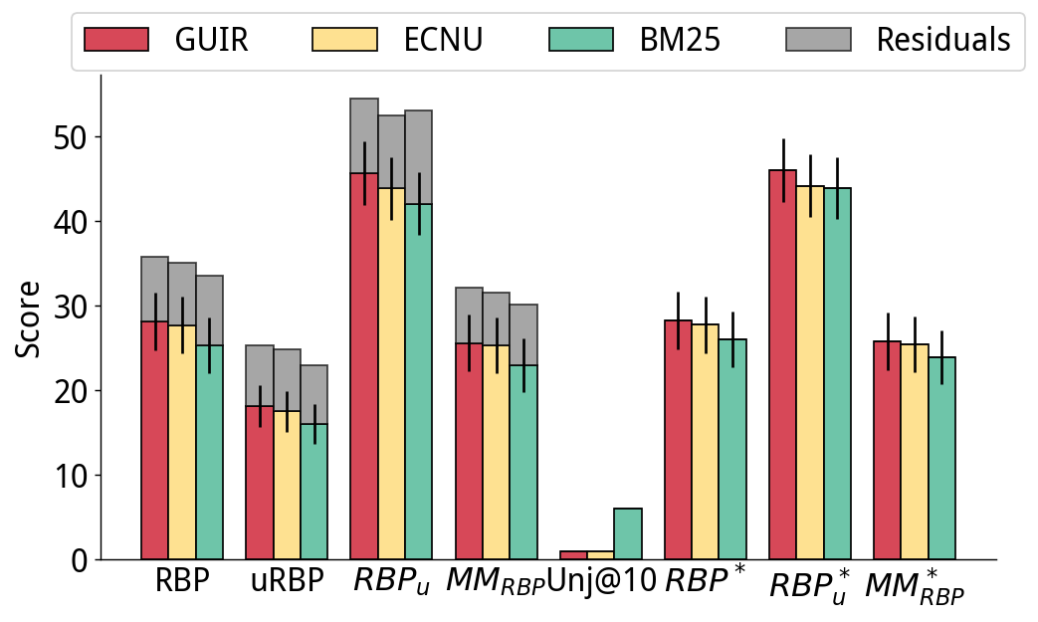
Baseline results for the best 2 submissions to Conference and Labs of the Evaluation Forum (CLEF) 2016 (Georgetown University Information Retrieval [GUIR] and East China Normal University [ECNU]) and the Best Match 25 (BM25) baseline of Terrier. MM: multidimensional metric; RBP: rank biased precision.

**Figure 5:**
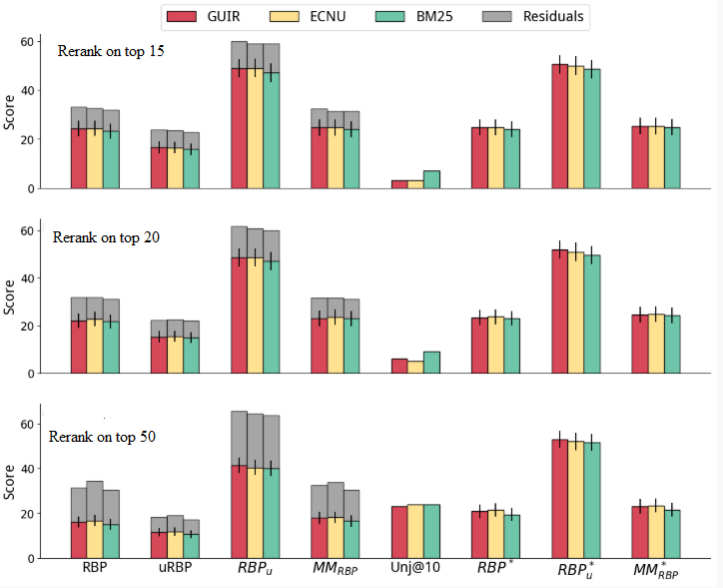
Reranking of the runs based on the Dale-Chall readability formula. ECNU: East China Normal University; GUIR: Georgetown University Information Retrieval; MM: multidimensional metric; RBP: rank biased precision.

**Figure 6:**
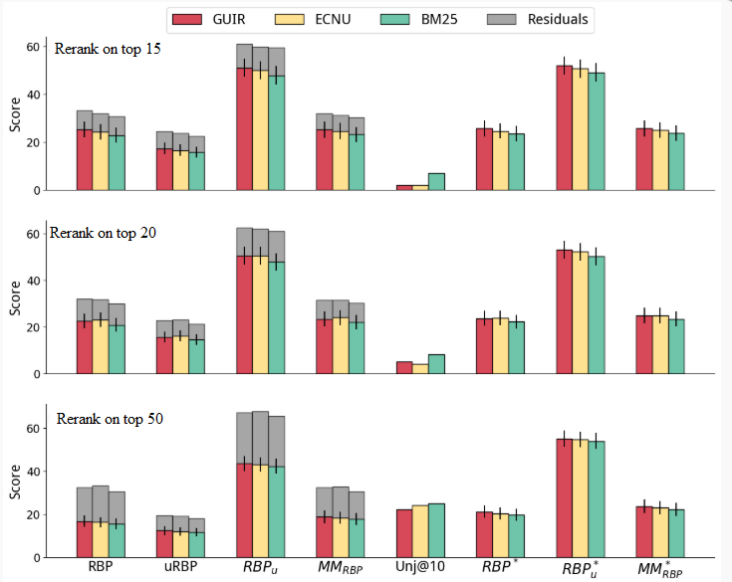
Reranking of the runs based on the eXtreme Gradient Boosting (XGB) regressor to estimate understandability. ECNU: East China Normal University; GUIR: Georgetown University Information Retrieval; MM: multidimensional metric; RBP: rank biased precision.

**Figure 7:**
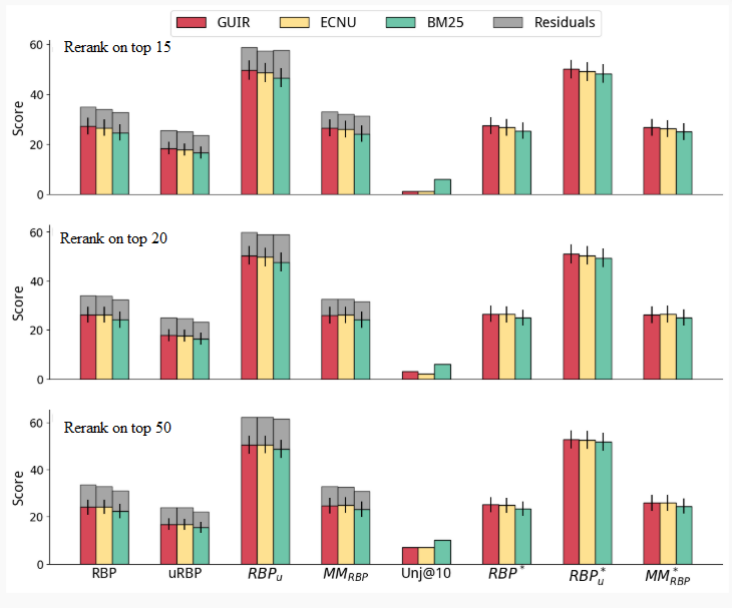
Reranking combining topical relevance (original run) and understandability (eXtreme Gradient Boosting [XGB]) through rank fusion. ECNU: East China Normal University; GUIR: Georgetown University Information Retrieval; MM: multidimensional metric; RBP: rank biased precision.

**Figure 8:**
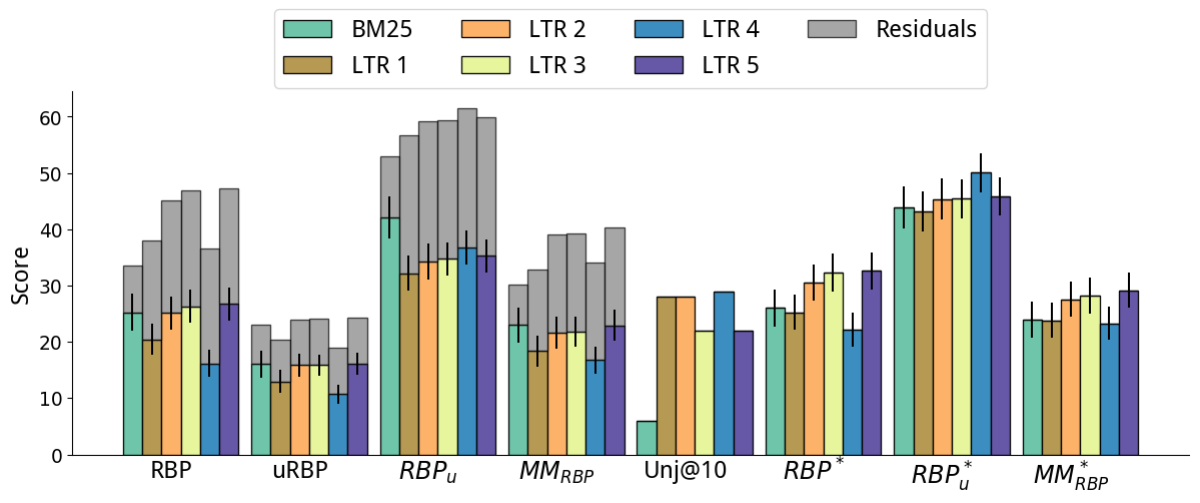
Results of the learning to rank (LTR) method on the Best Match 25 (BM25) baseline. The BM25 baseline (light blue) is shown for direct comparison. MM: multidimensional metric; RBP: rank biased precision.

Results for the considered retrieval methods are reported in Figures 4-8. We report only the results for CLEF 2016 for brevity; those for CLEF 2015 exhibited similar trends and are included in the Multimedia Appendix 5. When reranking results, we risk bringing to the top position a document that was never assessed. The RBP residuals (shown in gray in Figures 3-8) show the possible gains that unassessed documents can have on the evaluation, as it assumed that all unassessed documents are relevant. Another way to quantify the effect that unassessed documents have on evaluation is looking at the average number of unassessed documents in the top 10 results: this is given by the metric *Unj@10*. Larger values of *Unj@10* imply that actual effectiveness might be noticeably larger. Here, we also show the values for the condensed measures.

The effectiveness of the top 2 submissions to CLEF 2016 and the BM25 baseline are reported in Figure 4. In turn, we report the results of each subexperiment: *simple reranking* (Figures 5 and 6), *fusion experiments* (Figure 7), and *learning to rank* (Figure 8).

#### Simple Reranking

Figure 5 reports the results of reranking methods applied to the runs shown in Figure 4. Reranking was applied based on the DCI score of each document calculated using the preprocessing combination of Boilerpipe and ForcePeriod (best according to Pearson correlation, from Tables 3 and 4). We found that the relevance of the reranked runs (as measured by RBP_r_ and RBP^*^_r_) significantly decreased, compared with the original runs, for example, reranking the top 15 search results using DCI made RBP_r_ decrease from 25.28 to 21.58. However, as expected, these reranked results were significantly more understandable: for the previous example, RBP_u_ passed from 42.08 to 47.09.

In the experiments, we also studied the influence of the number of documents considered for reranking (cut-off). The top/middle/bottom plots of Figure 5 refer to reranking only the top k=15/20/50 documents from the original runs. The results show that the more documents are considered for reranking, the more degradation in RBP_r_ effectiveness. Considering understandability only in the evaluation shows mixed results. Similar trends were observed for evaluation measures that consider understandability (RBP and RBP_u_), however, with some exceptions. For example, an increase in uRBP was observed when reranking ECNU using the top 50 results.

Note that with the increase of the number of documents considered for reranking, there is an increase in the number of unassessed documents being considered by the evaluation measures. Nevertheless, we note that if unassessed documents are excluded from the evaluation, similar trends are observed, for example, compare findings with those for the condensed measures uRBP^*^, RBP^*^_r_, RBP^*^_u_, and MM^*^_RBP_.

Figure 6 refers to using a machine learning method, XGB regressor (Textbox 9), to estimate understandability. Similarly, when using DCI, as the cut-off increased, for example, from k=15 to k=50, the documents returned were more understandable but less relevant. For the same cut-off value, for example, k=15, the machine learning method used for estimating understandability consistently yielded more understandable results than DCI (higher RBP_u_ and RBP^*^_u_).

Overall, statistically significant improvements over the baselines were observed for most configurations and measures.

#### Rank Fusion

Next, we report the results of automatically combining topical relevance and understandability through rank fusion in Figure 7. We used the XGB method for estimating understandability, as it was the one yielding highest effectiveness for the reranking method. Runs were thus produced by fusing the reranking with XGB and the original run. (Results for DCI are reported in the Multimedia Appendix 5 and confirm the superiority of XGB.)

As for reranking, also for the rank fusion approaches we found that, in general, higher cut-offs were associated to higher effectiveness in terms of understandability measures on one hand, but higher losses in terms of relevance-oriented measures on the other. Overall, results obtained with rank fusion were superior to those obtained with reranking only, although most differences were not statistically significant. Statistically significant improvements over the baselines were instead observed for most configurations and measures.

#### Learning to Rank

Finally, we analyze the results obtained by the learning to rank methods in Figure 8. Unlike with the previous methods, we did not impose a rank cut-off on learning to rank. Learning to rank was only applied to the BM25 baseline, as we had no access to the IR features for the runs submitted at CLEF (ie, GUIR and ECNU for CLEF 2016). BM25 baseline (Figure 4) is also shown in Figure 8 for an easy and direct comparison.

When considering RBP_r_ and uRBP, learning to rank exhibited effectiveness that was significantly inferior to that of the GUIR and ECNU baseline runs, although higher than those for the BM25 baseline (for some configurations). The examination of the number of unassessed documents (and the RBP residuals, see Multimedia Appendix 5) revealed that this might have been because measures were affected by the large number of unassessed documents retrieved in the top 10 ranks. For example, the RBP_r_ residual for learning to rank methods was about double that of the baselines or other approaches (see Multimedia Appendix 5). In fact, among the documents retrieved in the top 10 results by learning to rank, there were 20% (2/10) that were unassessed, compared with an average of 3% (0.3/10) for the other methods (excluding XGB with cutoff 50, which also exhibited high residuals).

We thus should carefully account for unassessed documents through considering the residuals of RBP measures as well as the condensed measures. When this was done, we observed that learning to rank methods overall provided substantial gains over the original runs and other methods (when considering RBP^*^_r_, RBP^*^_u_, and MM^*^_RBP_), or large potential gains over these methods (when considering the residuals). Next, we analyzed these results in more detail.

No improvements over the baselines were found for LTR 1, and the high residuals for RBP_r_ were not matched by other residuals or by considering only assessed documents (see Multimedia Appendix 5). LTR 1 was a simple method that used only IR features and was trained only on topical relevance. Specifically, we devised 24 IR features using the Terrier framework. The score of various retrieval models was extracted from a multifield index composed of title, body, and whole document. Although simple, this is a typical learning to rank setting.

Compared with LTR 1, LTR 2 included the understandability features listed in Textboxes 1-10. This inclusion was as beneficial to the understandability measures as to the relevance measures, with RBP^*^_r_, RBP^*^_u_, and MM^*^_RBP_ all showing gains over the baselines. LTR 3 obtained similar MM^*^_RBP_ values, although with higher effectiveness for relevance measures (RBP^*^_r_) than for understandability (RBP^*^_u_).

LTRs 4 and 5 were devised based on a set understandability threshold U=40. Although LTR 4 took into consideration only documents that were easy to read (understandability label≤U), LTR 5 considered all documents, but boosted the relevance score. LTR 4 reached the highest understandability score for the learning-to-rank approaches (RBP^*^_u_=50.06), but it failed to retrieve a substantial number of relevant documents (RBP^*^_r_=2.20). In turn, LTR 5 reached the highest understandability-relevance trade-off (MM^*^_RBP_=29.20). Compared with the BM25 baseline (on which it was based), LTR 5 largely increased both relevance (RBP^*^_r_ from 26.01 to 32.60—a 25% increase, *P*_bl_=.003) and understandability (RBP^*^_u_ from 43.89 to 45.87 — a 4% increase, *P*_bl_<.001). Note that LTR 5 was also significantly better than the best run submitted to CLEF 2016 for both RBP^*^_r_ (15% increase, *P*_g_=.120) and MM^*^_RBP_ (13% increase, *P*_g_=.001).

## Discussion

### Principal Findings

The empirical experiments suggested the following:

- Machine learning methods based on regression are best suited to estimate the understandability of health Web pages
- Preprocessing does affect effectiveness (both for understandability prediction and document retrieval), although compared with other methods, ML-based methods for understandability estimation are less subjected to variability caused by poor preprocessing
- Learning to rank methods can be specifically trained to promote more understandable search results, whereas still providing an effective trade-off with topical relevance.

### Limitations

In this study, we relied on data collected through the CLEF 2015 and CLEF 2016 evaluation efforts to evaluate the effectiveness of methods that estimate the understandability of the Web pages. These assessments were obtained by asking medical experts and practitioners to rate documents; although, they were asked to estimate the understandability of the content as if they were the patients they treat, there might have been noise and imprecisions in the collection mechanism because of the subjectivity of the task. Figure 2 highlights this by showing that the agreement between assessors is relatively low. A better setting might have been to directly recruit health consumers: the task would still have been subjective but would have captured real ratings, rather than inferred or perceived ratings. Despite this, our previous work has shown that no substantial differences were found in the downstream evaluation of retrieval systems, when we acquired understandability assessments from health consumers for a subset of the CLEF 2015 collection [46].

Relevance assessments on the CLEF 2015 and 2016 collections are incomplete [44,14], that is, not all top ranked Web pages retrieved by the investigated methods have an explicit relevance assessment. This is often the case in information retrieval, where the validity of experiments based on incomplete assessments has been thoroughly investigated [88]. Nonetheless, we carefully controlled for the impact that unassessed documents had in our experiments by measuring their number and using measures such as RBP that account for residuals and condensed variants. The residuals analysis has been reported in the appendix.

### Conclusions

We have examined approaches to estimate the understandability of health Web pages, including the impact of HTML preprocessing techniques and how to integrate these within retrieval methods to provide more understandable search results for people seeking health information. We found that machine learning methods are better suited than traditionally employed readability measures for assessing the understandability of health-related Web pages and that learning to rank is the most effective strategy to integrate this into retrieval. We also found that HTML and text preprocessing do affect the effectiveness of both understandability estimations and of the retrieval process, although machine learning methods are less sensitive to this issue.

This paper contributes to improving search engines tailored to consumer health search because it thoroughly investigates promises and pitfalls of understandability estimations and their integration into retrieval methods. The paper further highlights which methods and settings should be used to provide better search results to health information seekers. As shown in Figure 1, these methods would clearly improve current health-focused search engines.

The methods investigated here do not provide a fully personalized search, with respect to how much of the health content consumers with different health knowledge might be able to understand. Instead, we focus on making the results understandable by anyone, and thus promote in the search results content that has the highest level of understandability. However, people with a more than average medical knowledge might benefit higher from more specialized content. We leave this personalization aspect, that is, the tailoring of the understandability level of the promoted content with respect to the user’s knowledge and abilities, to further work.

## Appendix

Multimedia Appendix 1 The impact of feature sets on the Spearman correlation between the predicted understandability and the ground truth assessed by human assessors in Conference and Labs of the Evaluation Forum (CLEF) eHealth 2015.

Multimedia Appendix 2 Distribution of Understandability Scores for Conference and Labs of the Evaluation Forum (CLEF) 2016.

Multimedia Appendix 3 Correlations between understandability estimators and human assessments for Conference and Labs of the Evaluation Forum (CLEF) 2015 and CLEF 2016.

Multimedia Appendix 4 Correlation results of different readability formulae with human assessments from Conference and Labs of the Evaluation Forum (CLEF) eHealth 2015 and 2016.

Multimedia Appendix 5 Results obtained by integrating understandability estimations within retrieval methods on Conference and Labs of the Evaluation Forum (CLEF) 2015 and CLEF 2016.

## Abbreviations

BM25: Best Match 25
CHV: consumer health vocabulary
CLEF: Conference and Labs of the Evaluation Forum
CLI: Coleman-Liau Index
CMV: consumer medical vocabulary
CRF: components of readability formulae
DCI: Dale-Chall Index
ECNU: East China Normal University
eHealth: electronic health
EMV: expert medical vocabulary
FRE: Flesch Reading Ease
GMV: general medical vocabulary
GUIR: Georgetown University Information Retrieval
HF: HTML features
HON: HealthOnNet
ICD: International Statistical Classification of Diseases and Related Health Problems
KISTI: Korean Institute of Science and Technology Information
LTR: learning to rank
MeSH: Medical Subject Headings
MLC: machine learning classifiers
MLR: machine learning regressors
MM: multidimensional metric
NLF: natural language features
OV: out-of-vocabulary
POS: part of speech
RBP: rank biased precision
RF: readability formulae
SMOG: Simple Measure of Gobbledygook
WFF: word frequency features
XGB: eXtreme Gradient Boosting
