# Supplementary material for: Consumer Health Search on the Web: Study of Web Page Understandability and Its Integration in Ranking Algorithms
Source: J Med Internet Res. 2019 Jan 30;21(1):e10986. doi: 10.2196/10986 (PMC6372940; doi:10.2196/10986)
Supplement: Multimedia Appendix 1 [file jmir_v21i1e10986_app1.pdf]

This is a Multimedia Appendix to a full manuscript published in the J Med Internet Res. For full copyright and citation information see http://dx.doi.org/10.2196/jmir.10986

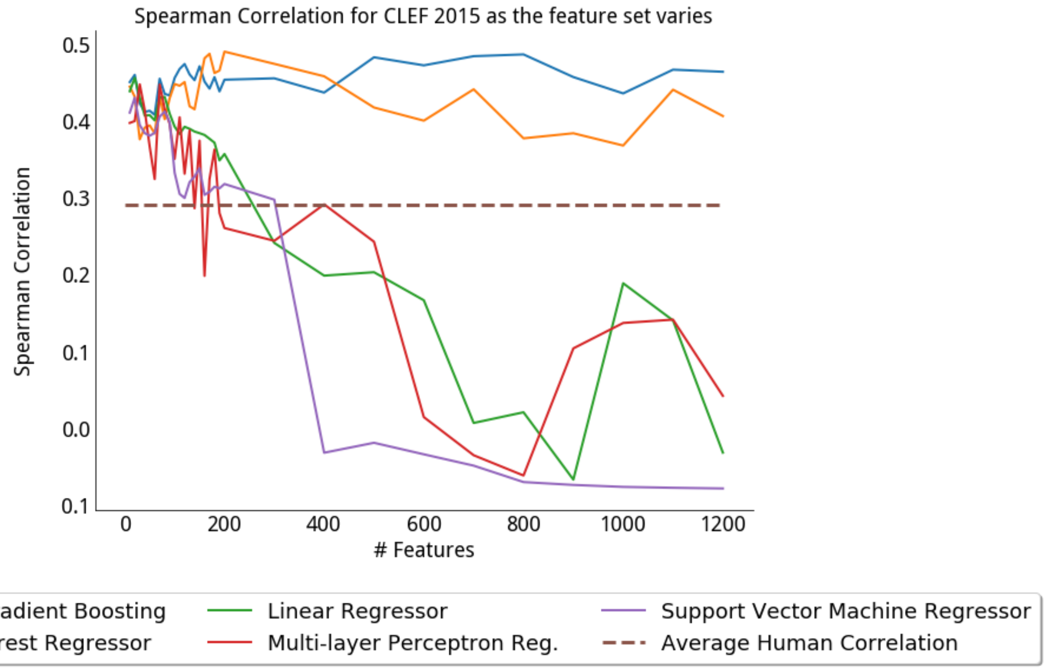

Figure 1. *To study the impact of feature sets, we measure the Spearman correlation between the predicted understandability and the ground truth assessed by human assessors in CLEF eHealth 2015. We varied the number of features each regressor algorithm was trained with. For that, we selected features with Python's f_regression algorithm from sklearn package. The eXtreme Gradient Boosting (XGB) and Random Forest Regressor were marginally influenced by the selection of features, therefore we used XGB with all features devised in our experiments.*
