# Supplementary material for: Consumer Health Search on the Web: Study of Web Page Understandability and Its Integration in Ranking Algorithms
Source: J Med Internet Res. 2019 Jan 30;21(1):e10986. doi: 10.2196/10986 (PMC6372940; doi:10.2196/10986)
Supplement: Multimedia Appendix 2 [file jmir_v21i1e10986_app2.pdf]

This is a Multimedia Appendix to a full manuscript published in the J Med Internet Res. For full copyright and citation information see http://dx.doi.org/10.2196/jmir.10986

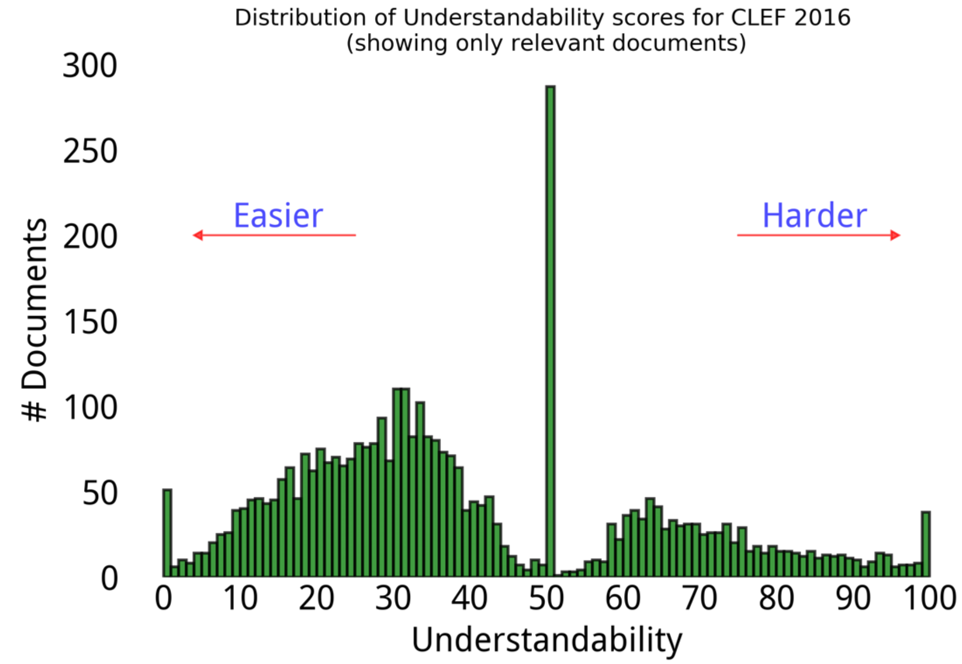

Figure 1. *Understandability label distribution for CLEF eHealth 2016.*

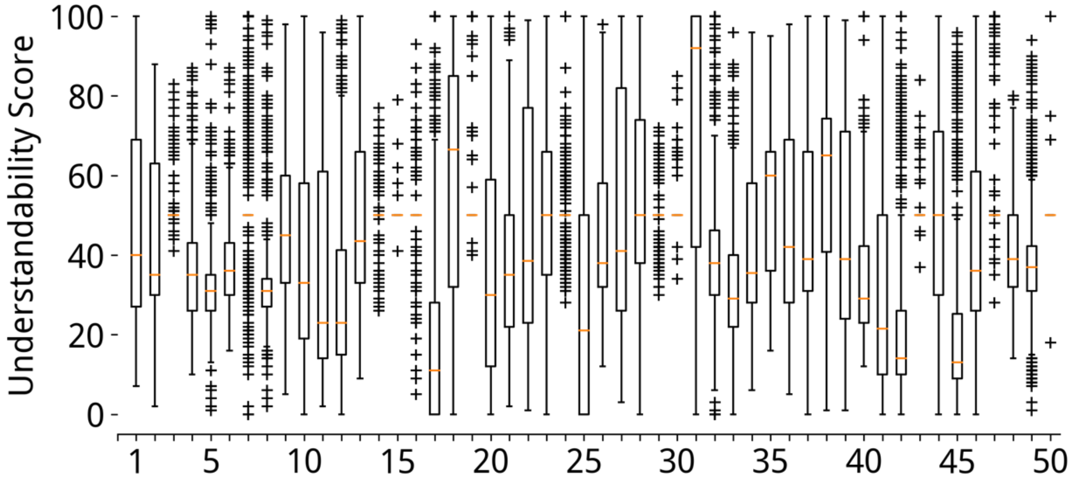

Figure 2. *Understandability distribution broken per topic for CLEF eHealth 2016.*
