# Supplementary figures and images for: Consumer Health Search on the Web: Study of Web Page Understandability and Its Integration in Ranking Algorithms
Source: J Med Internet Res. 2019 Jan 30;21(1):e10986. doi: 10.2196/10986 (PMC6372940; doi:10.2196/10986)

**Figure 1.** Correlations between understandability estimators and human assessments for CLEF 2015.

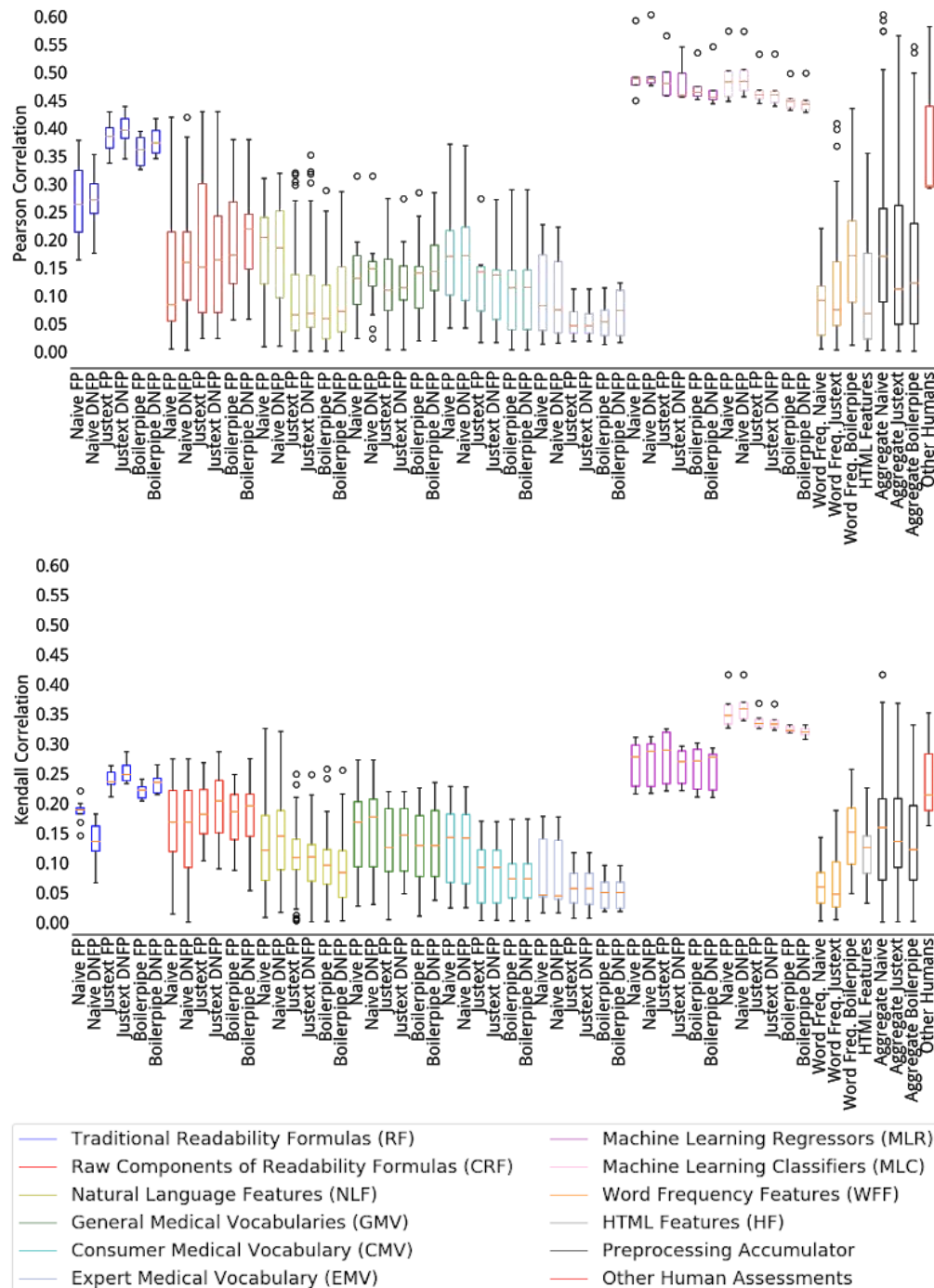

Supplement: Multimedia Appendix 3 [file jmir_v21i1e10986_app3.pdf]
