# Supplementary material for: Consumer Health Search on the Web: Study of Web Page Understandability and Its Integration in Ranking Algorithms
Source: J Med Internet Res. 2019 Jan 30;21(1):e10986. doi: 10.2196/10986 (PMC6372940; doi:10.2196/10986)
Supplement: Multimedia Appendix 4 [file jmir_v21i1e10986_app4.pdf]

This is a Multimedia Appendix to a full manuscript published in the J Med Internet Res. For full copyright and citation information see http://dx.doi.org/10.2196/jmir.10986

In Palotti et al. [1], authors investigate the influence of HTML preprocessing when readability formulas are used to estimate Webpage understandability. They found that readability formulas are heavily affected by the methods used to extract text from the HTML source, but they did not measure how correlated each method was with a human ground truth. We further extended Palotti et al.'s work to understand the influence of HTML preprocessing on automatic understandability methods and establish best practices. We show the correlation of each preprocessing combination with the ground truth assessments for CLEF 2015 and 2016.

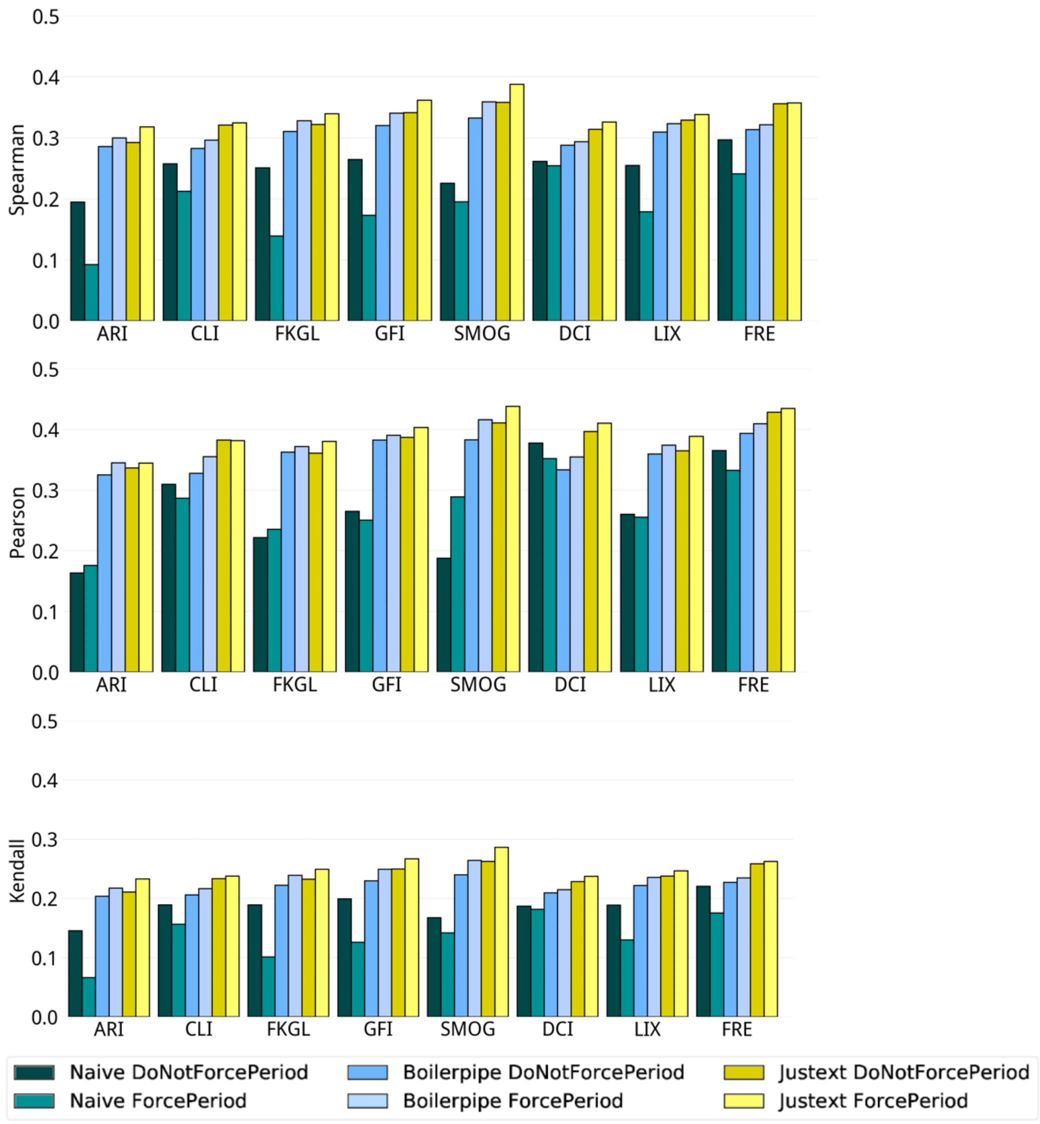

Figure 1. *Correlation results of different readability formulae with human assessments from CLEF eHealth 2015.*

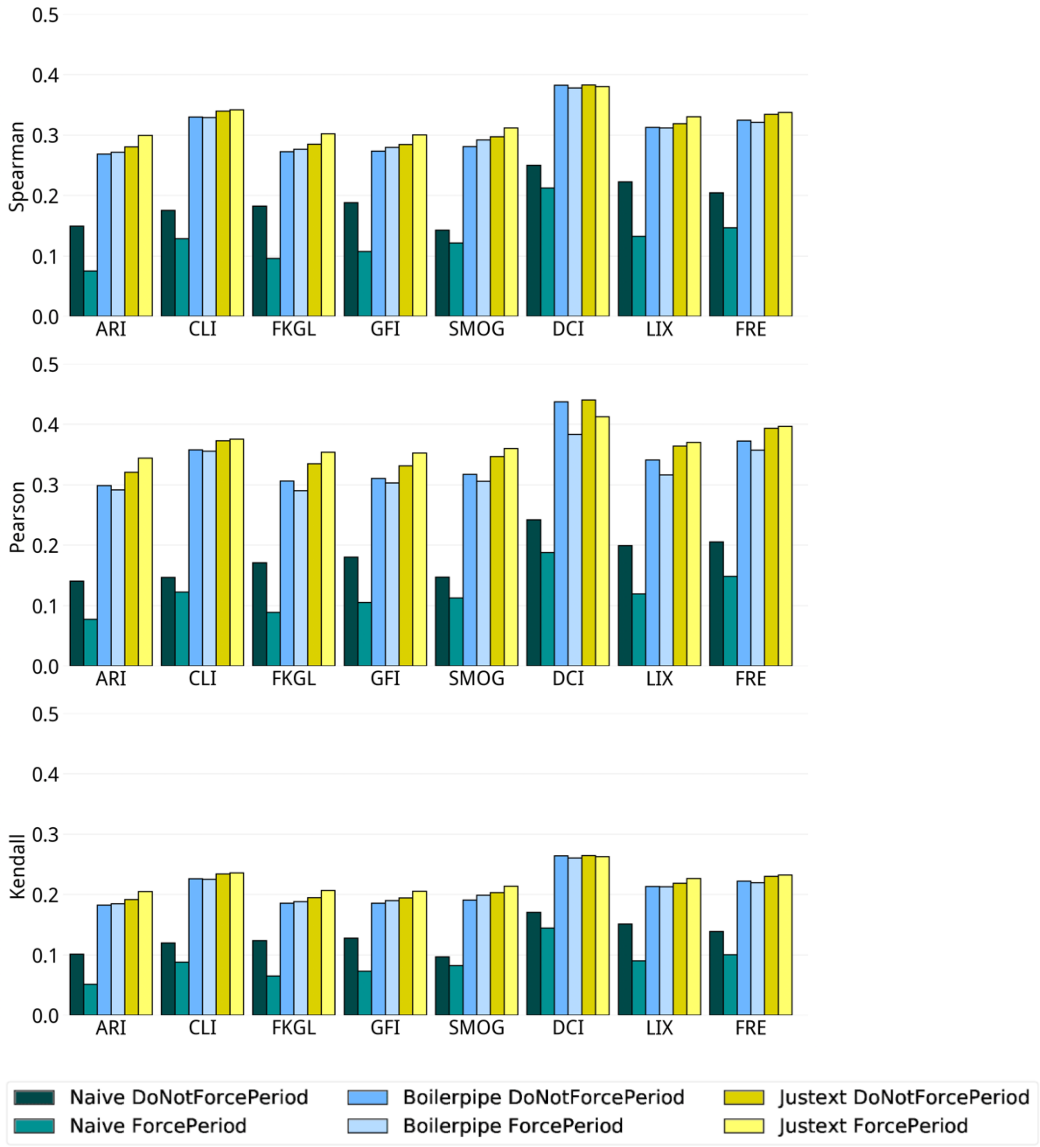

Figure 2. *Correlation results of different readability formulae with human assessments from CLEF eHealth 2016.*

[1] Palotti J, Zuccon G, Hanbury A. The Influence of Pre-processing on the Estimation of Readability of Web Documents. In: Proceedings of the 24th ACM International on Conference on Information and Knowledge Management. CIKM ’15. New York, NY, USA: ACM; 2015. p. 1763–1766. doi:10.1145/2806416.2806613.
