# Supplementary material for: Consumer Health Search on the Web: Study of Web Page Understandability and Its Integration in Ranking Algorithms
Source: J Med Internet Res. 2019 Jan 30;21(1):e10986. doi: 10.2196/10986 (PMC6372940; doi:10.2196/10986)
Supplement: Multimedia Appendix 5 [file jmir_v21i1e10986_app5.pdf]

This is a Multimedia Appendix to a full manuscript published in the J Med Internet Res. For full copyright and citation information see http://dx.doi.org/10.2196/jmir.10986

Idx	Rerank	Run	CLEF 2015 Measures				New Measures to Evaluate Understability in Retrieval							
			RBP_r	Res.	uRBP	Res.	RBP_u	Res.	MM_{RBP}	Res.	Unj@10	RBP_r^*	RBP_u^*	MM_{RBP}^*
1	No Rerank	ECNU (1st)	**51.57**	**8.95**	**50.51**	**8.95**	59.55	10.09	**46.22**	8.62	0.00	**51.57**	59.55	**46.22**
2	No Rerank	KISTI (2nd)	36.72$^{\diamond}$	8.06	35.92$^{\diamond}$	7.32	64.50	11.54	37.56$^{\diamond}$	7.89	**0.03**	37.07$^{\diamond}$	65.31	37.96$^{\diamond}$
3	No Rerank	BM25 Baseline	31.20$^{\diamond}$	8.76	30.51$^{\diamond}$	7.65	**67.60**	**12.20**	35.75$^{\diamond}$	**8.76**	**0.03**	31.57$^{\diamond}$	**68.94**$^{\diamond}$	36.42$^{\diamond}$
4	SMOG Top 15	Based on ECNU	38.16$^{\dagger\diamond}$	20.09	37.36$^{\dagger\diamond}$	8.95	55.11$^{\dagger\diamond}$	22.14	37.59$^{\dagger\diamond}$	18.98	0.14	45.45$^{\dagger\diamond}$	62.58	43.65
5	SMOG Top 15	Based on KISTI	31.28$^{\dagger\diamond}$	10.21	30.59$^{\dagger\diamond}$	7.48	67.05	13.95	34.23 $^{\diamond}$	10.05	0.05	33.12$^{\dagger\diamond}$	69.10$^{\diamond}$	36.02$^{\diamond}$
6	SMOG Top 15	Based on BM25	24.39$^{\dagger\diamond}$	11.46	23.83$^{\dagger\diamond}$	7.16	67.69	17.66	28.97$^{\dagger\diamond}$	11.46	0.10	26.28$^{\dagger\diamond}$	72.82$^{\dagger\diamond}$	31.53$^{\dagger\diamond}$
7	SMOG Top 20	Based on ECNU	34.88$^{\dagger\diamond}$	25.96	34.13$^{\dagger\diamond}$	8.95	54.15$^{\dagger\diamond}$	27.91	35.34$^{\dagger\diamond}$	25.02	0.20	44.87$^{\dagger\diamond}$	66.20$^{\dagger\diamond}$	44.88
8	SMOG Top 20	Based on KISTI	28.01$^{\dagger\diamond}$	11.05	27.39$^{\dagger\diamond}$	7.32	66.61	15.44	30.93$^{\dagger\diamond}$	10.82	0.08	30.87$^{\dagger\diamond}$	69.99$^{\dagger\diamond}$	33.81$^{\diamond}$
9	SMOG Top 20	Based on BM25	22.83$^{\dagger\diamond}$	13.48	22.31$^{\dagger\diamond}$	7.32	63.61	22.44	26.52$^{\dagger\diamond}$	13.48	0.15	26.22$^{\dagger\diamond}$	72.72$^{\dagger\diamond}$	30.87$^{\dagger\diamond}$
10	SMOG Top 50	Based on ECNU	21.83$^{\dagger\diamond}$	**36.63**	21.34$^{\dagger\diamond}$	8.95	41.71$^{\dagger\diamond}$	46.89	23.65$^{\dagger\diamond}$	**36.63**	**0.45**	39.88$^{\dagger\diamond}$	71.61$^{\dagger\diamond}$	43.86
11	SMOG Top 50	Based on KISTI	21.00$^{\dagger\diamond}$	18.67	20.53$^{\dagger\diamond}$	7.32	59.72	26.67	24.88$^{\dagger\diamond}$	18.67	0.23	27.20$^{\dagger\diamond}$	72.47$^{\dagger\diamond}$	31.90$^{\dagger\diamond}$
12	SMOG Top 50	Based on BM25	15.20$^{\dagger\diamond}$	18.73	14.86$^{\dagger\diamond}$	6.51	49.87$^{\dagger\diamond}$	36.41	17.79$^{\dagger\diamond}$	18.73	0.32	21.22$^{\dagger\diamond}$	73.17$^{\diamond}$	25.78$^{\dagger\diamond}$
13	XGB Top 15	Based on ECNU	**39.77**$^{\dagger\diamond}$	21.59	**38.90**$^{\dagger\diamond}$	**9.11**	54.84$^{\dagger\diamond}$	24.22	**38.35**$^{\dagger\diamond}$	20.72	0.15	**47.00**$^{\dagger\diamond}$	64.28$^{\dagger\diamond}$	45.22
14	XGB Top 15	Based on KISTI	31.36$^{\dagger\diamond}$	8.95	30.63$^{\dagger\diamond}$	6.83	**68.63**$^{\diamond}$	13.63	33.78$^{\diamond}$	8.78	0.05	32.80$^{\dagger\diamond}$	70.87$^{\dagger\diamond}$	35.24$^{\diamond}$
15	XGB Top 15	Based on BM25	23.38$^{\dagger\diamond}$	11.36	22.84$^{\dagger\diamond}$	7.16	66.79	19.43	27.60$^{\dagger\diamond}$	11.36	0.10	26.35$^{\dagger\diamond}$	73.48$^{\dagger\diamond}$	31.35$^{\dagger\diamond}$
16	XGB Top 20	Based on ECNU	34.91$^{\dagger\diamond}$	27.19	34.12$^{\dagger\diamond}$	**9.11**	52.69$^{\dagger\diamond}$	30.19	34.89$^{\dagger\diamond}$	25.95	0.25	46.71$^{\dagger\diamond}$	67.26$^{\dagger\diamond}$	**45.81**
17	XGB Top 20	Based on KISTI	29.04$^{\dagger\diamond}$	10.10	28.35$^{\dagger\diamond}$	6.51	69.10$^{\diamond}$	15.86	32.00$^{\dagger\diamond}$	9.94	0.07	31.82$^{\diamond}$	73.19$^{\dagger\diamond}$	34.84$^{\diamond}$
18	XGB Top 20	Based on BM25	21.83$^{\dagger\diamond}$	12.88	21.32$^{\dagger\diamond}$	6.51	64.16	25.29	25.47$^{\dagger\diamond}$	12.88	0.16	26.56$^{\dagger\diamond}$	76.26$^{\dagger\diamond}$	31.45$^{\dagger\diamond}$
19	XGB Top 50	Based on ECNU	22.75$^{\dagger\diamond}$	33.65	22.22$^{\dagger\diamond}$	8.79	43.36$^{\dagger\diamond}$	**49.18**	24.90$^{\dagger\diamond}$	33.65	**0.45**	40.69$^{\dagger\diamond}$	74.39$^{\dagger\diamond}$	44.65
20	XGB Top 50	Based on KISTI	18.69$^{\dagger\diamond}$	17.21	18.25$^{\dagger\diamond}$	6.67	56.87†	33.37	21.82$^{\dagger\diamond}$	17.21	0.29	27.69$^{\dagger\diamond}$	76.44$^{\dagger\diamond}$	32.53$^{\diamond}$
21	XGB Top 50	Based on BM25	17.47$^{\dagger\diamond}$	20.95	17.00$^{\dagger\diamond}$	6.02	47.21$^{\dagger\diamond}$	43.81	19.22$^{\dagger\diamond}$	20.98	0.41	27.09$^{\diamond}$	**79.77**$^{\dagger\diamond}$	31.35$^{\diamond}$
22	RRF (XGB & Orig.) Top 15	Based on ECNU	**47.81**$^{\dagger\diamond}$	12.85	**46.78**$^{\dagger\diamond}$	8.95	60.04	15.04	**44.69**	12.34	0.10	**50.09**	62.98$^{\dagger\diamond}$	46.85
23	RRF (XGB & Orig.) Top 15	Based on KISTI	33.78$^{\dagger\diamond}$	7.71	33.02$^{\dagger\diamond}$	6.83	68.57$^{\dagger\diamond}$	12.02	35.72†	7.55	0.03	34.34$^{\dagger\diamond}$	69.67$^{\dagger\diamond}$	36.26†
24	RRF (XGB & Orig.) Top 15	Based on BM25	26.85$^{\dagger\diamond}$	12.13	26.23$^{\dagger\diamond}$	7.48	66.64	15.98	31.52$^{\dagger\diamond}$	12.13	0.07	28.35$^{\dagger\diamond}$	70.65$^{\diamond}$	33.70$^{\dagger\diamond}$
25	RRF (XGB & Orig.) Top 20	Based on ECNU	46.49$^{\dagger\diamond}$	15.16	45.48$^{\dagger\diamond}$	9.11	59.95	17.12	43.69	14.54	0.12	49.95	64.30$^{\dagger\diamond}$	47.01
26	RRF (XGB & Orig.) Top 20	Based on KISTI	32.72$^{\dagger\diamond}$	8.45	31.97$^{\dagger\diamond}$	7.00	**69.06**$^{\dagger\diamond}$	12.77	35.14$^{\diamond}$	8.28	0.04	33.83$^{\dagger\diamond}$	70.83$^{\dagger\diamond}$	36.29$^{\diamond}$
27	RRF (XGB & Orig.) Top 20	Based on BM25	25.64$^{\dagger\diamond}$	12.51	25.05$^{\dagger\diamond}$	7.16	66.55	17.31	30.14$^{\dagger\diamond}$	12.51	0.09	27.64$^{\dagger\diamond}$	71.79$^{\dagger\diamond}$	32.96$^{\dagger\diamond}$
28	RRF (XGB & Orig.) Top 50	Based on ECNU	38.97$^{\dagger\diamond}$	**21.40**	38.08$^{\dagger\diamond}$	8.79	57.37	25.44	39.54$^{\dagger\diamond}$	19.83	**0.24**	47.00$^{\dagger\diamond}$	67.95$^{\dagger\diamond}$	**47.12**
29	RRF (XGB & Orig.) Top 50	Based on KISTI	27.78$^{\dagger\diamond}$	11.24	27.13$^{\dagger\diamond}$	6.83	67.83	16.75	31.07$^{\dagger\diamond}$	11.24	0.09	31.33$^{\dagger\diamond}$	**73.06**$^{\dagger\diamond}$	34.85$^{\diamond}$
30	RRF (XGB & Orig.) Top 50	Based on BM25	19.28$^{\dagger\diamond}$	17.11	18.86$^{\dagger\diamond}$	7.00	57.40†	**27.02**	22.78$^{\dagger\diamond}$	17.11	0.19	25.06$^{\dagger\diamond}$	71.56$^{\diamond}$	30.26$^{\dagger\diamond}$
31	RRF (SMOG & Orig.) Top 15	Based on ECNU	47.23$^{\dagger\diamond}$	12.41	46.24$^{\dagger\diamond}$	8.95	59.57	13.88	43.96$^{\dagger\diamond}$	11.80	0.08	49.21$^{\dagger\diamond}$	61.45	45.42
32	RRF (SMOG & Orig.) Top 15	Based on KISTI	34.84$^{\diamond}$	8.34	34.08$^{\diamond}$	7.48	67.87$^{\dagger\diamond}$	11.84	36.86$^{\diamond}$	8.18	0.03	35.32$^{\diamond}$	68.84$^{\dagger\diamond}$	37.37$^{\diamond}$
33	RRF (SMOG & Orig.) Top 15	Based on BM25	27.16$^{\dagger\diamond}$	10.79	26.55$^{\dagger\diamond}$	7.32	66.44	14.65	32.24$^{\dagger\diamond}$	10.79	0.05	28.21$^{\dagger\diamond}$	69.38$^{\diamond}$	33.67$^{\dagger\diamond}$
34	RRF (SMOG & Orig.) Top 20	Based on ECNU	45.91$^{\dagger\diamond}$	14.14	44.94$^{\dagger\diamond}$	9.11	59.46	15.28	43.50$^{\dagger\diamond}$	13.55	0.09	48.67$^{\dagger\diamond}$	62.46$^{\dagger\diamond}$	45.93
35	RRF (SMOG & Orig.) Top 20	Based on KISTI	34.17$^{\diamond}$	8.54	33.42$^{\diamond}$	7.48	68.23$^{\dagger\diamond}$	12.09	36.23$^{\diamond}$	8.38	0.03	34.79$^{\diamond}$	69.30$^{\dagger\diamond}$	36.89$^{\diamond}$
36	RRF (SMOG & Orig.) Top 20	Based on BM25	25.80$^{\dagger\diamond}$	12.33	25.22$^{\dagger\diamond}$	7.48	64.98	16.18	30.69$^{\dagger\diamond}$	12.33	0.08	27.24$^{\dagger\diamond}$	69.14$^{\diamond}$	32.68$^{\dagger\diamond}$
37	RRF (SMOG & Orig.) Top 50	Based on ECNU	39.06$^{\dagger\diamond}$	20.77	38.20$^{\dagger\diamond}$	**9.27**	57.68	23.16	39.55$^{\dagger\diamond}$	**20.28**	0.18	45.59$^{\dagger\diamond}$	65.82$^{\dagger\diamond}$	46.13
38	RRF (SMOG & Orig.) Top 50	Based on KISTI	29.51$^{\dagger\diamond}$	10.35	28.83$^{\dagger\diamond}$	7.48	68.60$^{\dagger\diamond}$	14.71	33.37	10.35	0.08	31.48$^{\dagger\diamond}$	71.70$^{\dagger\diamond}$	35.54
39	RRF (SMOG & Orig.) Top 50	Based on BM25	18.70$^{\dagger\diamond}$	17.12	18.29$^{\dagger\diamond}$	7.16	57.50†	24.59	22.73$^{\dagger\diamond}$	17.12	0.15	22.95$^{\dagger\diamond}$	68.61	28.21$^{\dagger\diamond}$
40	XGB LeToR	LTR 1 on BM25	24.86$^{\dagger\diamond}$	17.39	24.32$^{\dagger\diamond}$	7.81	**55.60**$^{\dagger\diamond}$	24.11	28.89$^{\dagger\diamond}$	17.39	0.22	29.67$^{\diamond}$	**66.41**	34.76$^{\diamond}$
41	XGB LeToR	LTR 2 on BM25	**30.72**$^{\diamond}$	21.25	**30.08**$^{\diamond}$	8.46	48.87$^{\dagger\diamond}$	28.82	**31.76**$^{\diamond}$	18.99	0.26	37.09$^{\diamond}$	61.89†	39.17$^{\diamond}$
42	XGB LeToR	LTR 3 on BM25	28.92$^{\diamond}$	**24.35**	28.32$^{\diamond}$	8.46	49.02$^{\dagger\diamond}$	32.11	30.14$^{\dagger\diamond}$	23.83	0.31	**37.32**$^{\diamond}$	63.86†	**39.84**$^{\diamond}$
43	XGB LeToR	LTR 4 on BM25	25.65$^{\dagger\diamond}$	25.72	25.09*	8.30	49.00$^{\dagger\diamond}$	**33.39**	27.45$^{\dagger\diamond}$	**24.40**	**0.33**	35.82$^{\diamond}$	66.14	38.21$^{\diamond}$
44	XGB LeToR	LTR 5 on BM25	30.21$^{\diamond}$	20.79	29.59$^{\diamond}$	**8.62**	48.47$^{\dagger\diamond}$	27.88	30.95$^{\dagger\diamond}$	19.99	0.25	37.11$^{\diamond}$	61.25$^{\dagger\diamond}$	39.15$^{\diamond}$

Table 1. *Results obtained by integrating understandability estimations within retrieval methods on CLEF 2015. Baseline runs are reported at table indices 1–3 (the index column is labeled Idx). Re-ranking experiments are reported at indices 4–21. Fusion experiments are reported at indices 22–39. Learning to rank experiments are reported at indices 40–44. All measures were calculated up to rank n = 10. The highest result of each set of experiments is reported in boldface for each measure. Statistically significant differences compared to ECNU are indicated with ⋄, while differences between an original run (indices 1-3) and its modifications are indicated with † (paired, two-tail t-test, $P < 0.05$).*

Idx	Rerank	Run	CLEF 2016 Measures				New Measures to Evaluate Understability in Retrieval							
			RBP_r	Res.	uRBP	Res.	RBP_u	Res.	MM_{RBP}	Res.	Unj@10	RBP^*_r	RBP^*_u	MM^*_{RBP}
1	No Rerank	GUIR (1st)	**28.11**	7.65	**18.12**	7.19	**45.69**	8.86	**25.61**	6.50	0.01	**28.29**	**46.03**	**25.79**
2		ECNU (2nd)	27.70	7.37	17.55	**7.34**	43.89$^{\diamond}$	8.66	25.35	6.26	0.01	27.77	44.18$^{\diamond}$	25.48
3		BM25 Baseline	25.28$^{\diamond}$	**8.24**	16.05$^{\diamond}$	6.94	42.08$^{\diamond}$	**10.97**	22.97$^{\diamond}$	**7.19**	**0.06**	26.01$^{\diamond}$	43.89$^{\diamond}$	23.93$^{\diamond}$
4	Dale-Chall Top 15	Based on GUIR	24.70$^{\dagger\diamond}$	8.70	16.83$^{\dagger\diamond}$	7.27	49.10$^{\dagger\diamond}$	10.62	24.94	7.50	0.03	25.24$^{\dagger\diamond}$	50.33$^{\dagger\diamond}$	25.54
5		Based on ECNU	24.78$^{\dagger\diamond}$	7.83	16.64$^{\diamond}$	7.16	48.88$^{\dagger\diamond}$	9.71	24.80	6.50	0.02	25.12$^{\dagger\diamond}$	49.64$^{\dagger\diamond}$	25.21
6		Based on BM25	23.22$^{\dagger\diamond}$	8.78	15.85 $^{\diamond}$	6.94	47.09$^{\dagger\diamond}$	11.83	24.01	7.42	0.07	24.04$^{\dagger\diamond}$	48.60$^{\dagger\diamond}$	24.82
7	Dale-Chall Top 20	Based on GUIR	22.19$^{\dagger\diamond}$	9.37	15.36$^{\dagger\diamond}$	6.98	48.71$^{\dagger\diamond}$	12.30	23.21$^{\dagger\diamond}$	8.12	0.06	23.26$^{\dagger\diamond}$	51.39$^{\dagger\diamond}$	24.45$^{\dagger\diamond}$
8		Based on ECNU	23.01$^{\dagger\diamond}$	8.93	15.70$^{\dagger\diamond}$	6.91	48.99$^{\dagger\diamond}$	11.69	23.73$^{\dagger\diamond}$	7.80	0.05	23.84$^{\dagger\diamond}$	51.00$^{\dagger\diamond}$	24.66
9		Based on BM25	21.58$^{\dagger\diamond}$	9.51	14.83$^{\dagger\diamond}$	7.02	46.99†	13.00	22.89$^{\diamond}$	8.06	0.09	22.93$^{\dagger\diamond}$	49.55$^{\dagger\diamond}$	24.26
10	Dale-Chall Top 50	Based on GUIR	16.18$^{\dagger\diamond}$	15.24	11.56$^{\dagger\diamond}$	6.80	41.79$^{\dagger\diamond}$	24.49	18.10$^{\dagger\diamond}$	14.42	0.22	20.90$^{\dagger\diamond}$	53.28$^{\dagger\diamond}$	23.27$^{\dagger\diamond}$
11		Based on ECNU	16.88$^{\dagger\diamond}$	17.37	11.78$^{\dagger\diamond}$	**7.30**	40.76$^{\dagger\diamond}$	23.77	18.30$^{\dagger\diamond}$	**15.57**	**0.24**	21.34$^{\dagger\diamond}$	52.07$^{\dagger\diamond}$	23.33$^{\dagger\diamond}$
12		Based on BM25	15.06$^{\dagger\diamond}$	15.35$^{\dagger\diamond}$	10.55	6.62	40.03 $^{\diamond}$	23.88	16.55$^{\dagger\diamond}$	13.83	**0.24**	19.42$^{\dagger\diamond}$	51.69$^{\dagger\diamond}$	21.59$^{\dagger\diamond}$
13	XGB Top 15	Based on GUIR	**25.16**$^{\dagger\diamond}$	8.09	**17.27**$^{\dagger\diamond}$	7.12	**50.96**$^{\dagger\diamond}$	10.11	**25.16**	6.89	0.02	**25.61**$^{\dagger\diamond}$	52.00$^{\dagger\diamond}$	**25.68**
14		Based on ECNU	24.18$^{\dagger\diamond}$	7.69	16.54 $^{\diamond}$	7.09	50.00$^{\dagger\diamond}$	9.91	24.56	6.65	0.02	24.56$^{\dagger\diamond}$	50.74$^{\dagger\diamond}$	25.01
15		Based on BM25	22.33$^{\dagger\diamond}$	8.14	15.46	6.76	47.90$^{\dagger\diamond}$	12.13	22.89$^{\diamond}$	7.25	0.07	23.11$^{\dagger\diamond}$	49.43$^{\dagger\diamond}$	23.69$^{\diamond}$
16	XGB Top 20	Based on GUIR	22.38$^{\dagger\diamond}$	9.49	15.61$^{\dagger\diamond}$	7.05	50.45$^{\dagger\diamond}$	12.08	23.30$^{\dagger\diamond}$	8.16	0.05	23.62$^{\dagger\diamond}$	52.98$^{\dagger\diamond}$	24.68
17		Based on ECNU	22.95$^{\dagger\diamond}$	8.82	15.95$^{\dagger\diamond}$	7.02	50.42$^{\dagger\diamond}$	11.70	23.97$^{\diamond}$	7.56	0.04	23.68$^{\dagger\diamond}$	52.15$^{\dagger\diamond}$	24.73
18		Based on BM25	20.65$^{\dagger\diamond}$	9.42	14.46$^{\dagger\diamond}$	6.84	47.74$^{\dagger\diamond}$	13.56	21.93$^{\diamond}$	8.34	0.09	21.98$^{\dagger\diamond}$	50.28$^{\dagger\diamond}$	23.27$^{\diamond}$
19	XGB Top 50	Based on GUIR	16.65$^{\dagger\diamond}$	15.73	12.39$^{\dagger\diamond}$	6.84	43.49$^{\dagger\diamond}$	23.63	18.70$^{\dagger\diamond}$	13.74	0.22	21.13$^{\dagger\diamond}$	**55.07**$^{\dagger\diamond}$	23.58$^{\dagger\diamond}$
20		Based on ECNU	16.19$^{\dagger\diamond}$	**17.01**	11.82$^{\dagger\diamond}$	7.27	43.05$^{\diamond}$	**24.75**	18.27$^{\dagger\diamond}$	14.41	**0.24**	20.16$^{\dagger\diamond}$	54.70$^{\dagger\diamond}$	22.96$^{\dagger\diamond}$
21		Based on BM25	15.43$^{\dagger\diamond}$	15.37	11.33$^{\dagger\diamond}$	6.48	41.93$^{\diamond}$	23.65	17.43$^{\dagger\diamond}$	13.40	0.26	19.58$^{\dagger\diamond}$	54.04$^{\dagger\diamond}$	22.17$^{\dagger\diamond}$
22	RRF (XGB & Orig.) Top 15	Based on GUIR	**27.23**$^{\dagger\diamond}$	7.76	**18.31**	7.23	49.69$^{\dagger\diamond}$	9.18	26.49$^{\dagger\diamond}$	6.62	0.01	**27.46**$^{\dagger\diamond}$	50.07$^{\dagger\diamond}$	**26.69**$^{\dagger\diamond}$
23		Based on ECNU	26.60$^{\dagger\diamond}$	7.41	17.81	7.19	48.67$^{\dagger\diamond}$	8.80	26.02	6.09	0.01	26.76$^{\dagger\diamond}$	49.10$^{\dagger\diamond}$	26.27†
24		Based on BM25	24.57$^{\diamond}$	8.15	16.51$^{\diamond}$	6.91	46.76†	11.23	24.16†	7.20	0.06	25.32$^{\diamond}$	48.52$^{\dagger\diamond}$	25.08†
25	RRF (XGB & Orig.) Top 20	Based on GUIR	26.21$^{\dagger\diamond}$	7.96	17.73	7.19	50.29$^{\dagger\diamond}$	9.58	25.89	6.73	0.03	26.53$^{\dagger\diamond}$	50.98$^{\dagger\diamond}$	26.25
26		Based on ECNU	26.15$^{\dagger\diamond}$	7.64	17.69	7.09	49.70$^{\dagger\diamond}$	9.28	26.07	6.39	0.02	26.38$^{\dagger\diamond}$	50.32$^{\dagger\diamond}$	26.35
27		Based on BM25	24.04$^{\dagger\diamond}$	8.24	16.32$^{\diamond}$	6.87	47.69$^{\dagger\diamond}$	11.40	24.08$^{\dagger\diamond}$	7.35	0.06	24.82$^{\dagger\diamond}$	49.52$^{\dagger\diamond}$	25.01†
28	RRF (XGB & Orig.) Top 50	Based on GUIR	24.09$^{\dagger\diamond}$	9.44	16.85$^{\dagger\diamond}$	7.02	50.55$^{\dagger\diamond}$	11.76	24.76	8.01	0.07	25.08$^{\dagger\diamond}$	**52.84**$^{\dagger\diamond}$	25.84
29		Based on ECNU	24.17$^{\dagger\diamond}$	8.67	16.75$^{\diamond}$	7.12	**50.63**$^{\dagger\diamond}$	11.66	25.00	7.61	0.07	24.90$^{\dagger\diamond}$	52.50$^{\dagger\diamond}$	25.84
30		Based on BM25	22.28$^{\dagger\diamond}$	8.87	15.50	6.76	48.79$^{\dagger\diamond}$	**12.90**	23.13$^{\dagger\diamond}$	7.82	**0.10**	23.46$^{\dagger\diamond}$	51.89$^{\dagger\diamond}$	24.57
31	RRF (Dale-Chall & Orig.) Top 15	Based on GUIR	26.79$^{\dagger\diamond}$	8.06	17.94	7.37	48.43$^{\dagger\diamond}$	9.34	26.16	6.81	0.02	27.06$^{\dagger\diamond}$	48.98$^{\dagger\diamond}$	26.46
32		Based on ECNU	26.81	7.58	17.70	7.30	47.98$^{\dagger\diamond}$	9.04	26.25	6.30	0.02	27.00	48.53$^{\dagger\diamond}$	26.50†
33		Based on BM25	24.91$^{\diamond}$	8.12	16.58$^{\diamond}$	7.09	45.85†	10.75	24.67†	6.99	0.06	25.68$^{\diamond}$	47.55$^{\dagger\diamond}$	25.56†
34	RRF (Dale-Chall & Orig.) Top 20	Based on GUIR	25.91$^{\dagger\diamond}$	8.20	17.49$^{\dagger\diamond}$	7.27	48.76$^{\dagger\diamond}$	9.92	25.75	6.93	0.03	26.31$^{\dagger\diamond}$	49.66$^{\dagger\diamond}$	26.19
35		Based on ECNU	26.38$^{\dagger\diamond}$	8.03	17.50	**7.44**	48.55$^{\dagger\diamond}$	9.50	**26.25**	6.72	0.03	26.64$^{\dagger\diamond}$	49.37$^{\dagger\diamond}$	26.57$^{\diamond}$
36		Based on BM25	24.73$^{\diamond}$	8.21	16.63$^{\diamond}$	7.12	46.63†	11.00	24.85†	7.11	0.06	25.51$^{\diamond}$	48.44$^{\dagger\diamond}$	25.77†
37	RRF (Dale-Chall & Orig.) Top 50	Based on GUIR	23.29$^{\dagger\diamond}$	**9.76**	16.15$^{\dagger\diamond}$	7.12	49.04$^{\dagger\diamond}$	12.47	24.13	**8.37**	0.07	24.48$^{\dagger\diamond}$	51.45$^{\dagger\diamond}$	25.41
38		Based on ECNU	24.16$^{\dagger\diamond}$	9.12	16.37$^{\diamond}$	7.34	48.84$^{\dagger\diamond}$	11.64	24.78	7.86	0.07	25.06$^{\dagger\diamond}$	50.88$^{\dagger\diamond}$	25.80
39		Based on BM25	22.64$^{\dagger\diamond}$	9.26	15.47$^{\diamond}$	7.16	47.25$^{\dagger\diamond}$	12.79	23.67	7.86	0.09	23.89$^{\dagger\diamond}$	50.26$^{\dagger\diamond}$	25.11
40	XGB LeToR	LTR 1 on BM25	20.42$^{\dagger\diamond}$	17.61	13.00$^{\dagger\diamond}$	7.41	32.17$^{\dagger\diamond}$	24.61	18.39$^{\dagger\diamond}$	14.41	0.28	25.25$^{\diamond}$	43.19$^{\diamond}$	23.83$^{\diamond}$
41		LTR 2 on BM25	24.98$^{\dagger\diamond}$	19.83	15.30$^{\dagger\diamond}$	8.09	35.09$^{\dagger\diamond}$	25.14	22.26$^{\diamond}$	17.50	0.24	30.41	46.09	28.28$^{\dagger\diamond}$
42		LTR 3 on BM25	26.35†	**20.48**	15.88$^{\dagger\diamond}$	8.16	34.73$^{\dagger\diamond}$	24.69	21.81†	17.41	0.22	32.25$^{\diamond}$	45.44	28.22$^{\dagger\diamond}$
43		LTR 4 on BM25	16.16$^{\dagger\diamond}$	19.48	10.76$^{\dagger\diamond}$	7.27	**36.75**$^{\dagger\diamond}$	**28.51**	16.77$^{\dagger\diamond}$	**17.80**	**0.29**	22.20$^{\dagger\diamond}$	**50.06**$^{\dagger\diamond}$	23.32$^{\diamond}$
44		LTR 5 on BM25	**26.76**$^{\diamond}$	**20.48**	**16.19**$^{\diamond}$	**8.34**	35.26$^{\dagger\diamond}$	24.13	**22.96**	17.59	0.22	**32.60**†	45.87	**29.20**$^{\dagger\diamond}$

Table 2. *Results obtained by integrating understandability estimations within retrieval methods on CLEF 2016. Baseline runs are reported at table indices 1–3 (the index column is labeled Idx). Re-ranking experiments are reported at indices 4–21. Fusion experiments are reported at indices 22–39. Learning to rank experiments are reported at indices 40–44. All measures were calculated up to rank n = 10. The highest result of each set of experiments is reported in boldface for each measure. Statistically significant differences compared to ECNU are indicated with ⋄, while differences between an original run (indices 1-3) and its modifications are indicated with † (paired, two-tail t-test, $P < 0.05$).*
